# JAK/STAT-mediated regulation of PD-L1 expression in breast cancer: Molecular mechanisms and implications for immunotherapy

**DOI:** 10.1016/j.isci.2026.114826

**Published:** 2026-01-30

**Authors:** Hua Sang, Xudong Zhao

**Affiliations:** 1 Pharmacy Department, Zhongshan Hospital Wusong Branch, Fudan University (Shanghai Baoshan District Wusong Central Hospital), Baoshan, Shanghai 200940, China; 2 Affiliated Hospital of Nantong University, Nantong, Jiangsu 226000, China

**Keywords:** Biological sciences

## Abstract

Immune checkpoint blockade targeting the PD-1/PD-L1 axis has expanded treatment options for breast cancer, particularly triple-negative disease, yet therapeutic responses are limited by immune escape mechanisms. This review synthesizes mechanistic evidence showing how JAK/STAT signaling, predominantly through STAT1 and STAT3, integrates inflammatory cues, oncogenic stress, and microenvironmental signals to regulate PD-L1 expression across tumor cells and immune compartments. Experimental studies using cell lines, patient specimens, and *in vivo* models reveal that cytokines, hypoxia, metabolic remodeling, and extracellular vesicles sustain STAT-dependent PD-L1 programs that dampen cytotoxic immunity and promote resistance to therapy. The review further examines pharmacologic, genetic, and nanotechnology-based strategies that disrupt this signaling axis and enhance immune checkpoint efficacy, including combination regimens with chemotherapy, targeted therapy, and photodynamic approaches. Collectively, these findings position JAK/STAT-driven PD-L1 regulation as a unifying framework for understanding immune evasion and optimizing immunotherapy in breast cancer.

## Graphical abstract

**Figure:**
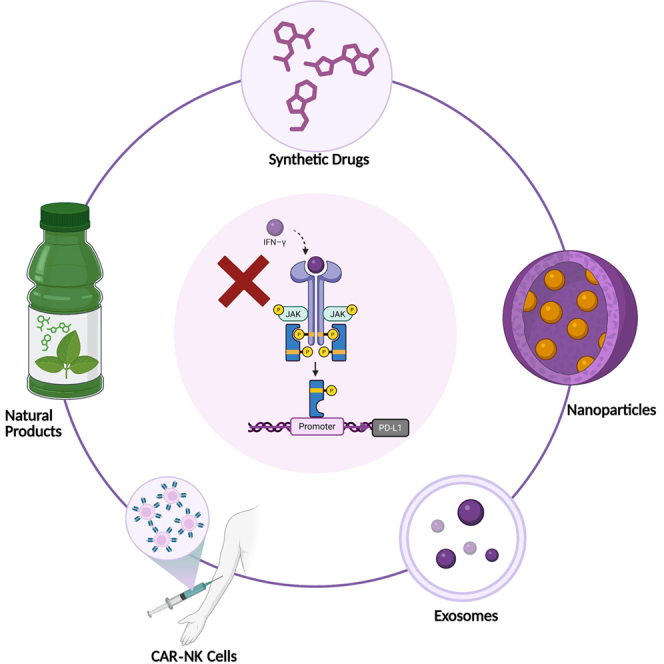

## Introduction

Breast cancer, the most common malignancy worldwide, displays substantial heterogeneity that arises from a complex interaction of genetic alterations, hormonal influences, and environmental or lifestyle-related factors.^1^^,^^2^ Breast cancer care is adapted to the tumor’s subtype and stage. Treatment usually starts with surgery and is supplemented with radiotherapy, chemotherapy, or a combined approach depending on clinical needs.^3^ Hormone receptor-positive breast cancers are treated with selective estrogen receptor modulators, estrogen receptor degraders, and aromatase inhibitors. Human epidermal growth factor receptor 2 (HER2)-positive tumors are managed with monoclonal antibodies, kinase inhibitors, and antibody-drug conjugates.^4^ Immunotherapy has emerged as a promising strategy in breast cancer because it engages the immune system to recognize and eliminate malignant cells. Although early efforts were directed at immunologically cold subtypes such as triple-negative breast cancer (TNBC), recent progress has broadened its application. In particular, the introduction of immune checkpoint inhibitors, such as pembrolizumab, has led to meaningful improvements in clinical outcomes when combined with chemotherapy.^5^ The development of antibody-drug conjugates, cancer vaccines, and bispecific antibodies has broadened the therapeutic landscape for hormone receptor-positive and HER2-negative, as well as HER2-positive, breast cancers. These approaches strengthen antitumor immunity by counteracting immunosuppressive mechanisms within the tumor microenvironment and provide targeted strategies to address therapeutic resistance. As a result, they contribute to more personalized and effective breast cancer management.^6^^,^^7^ Programmed death-ligand 1 (PD-L1) is an immune checkpoint protein highly expressed in breast cancer. It suppresses T cell activity by binding to PD-1 receptors, which helps tumors evade immune surveillance. PD-L1 is found on cancer cells and tumor-infiltrating immune cells. High PD-L1 expression on tumor cells usually indicates immune resistance, while its presence on immune cells may suggest an active immune response.^8^ PD-1/PD-L1-based immunotherapy has become a pivotal advancement in breast cancer treatment, restoring immune activity to detect and destroy cancer cells.^9^ These treatments are particularly effective in aggressive forms such as TNBC. They function by blocking the interaction between PD-1 receptors on T cells and PD-L1 on tumor cells, thereby disrupting immune escape mechanisms and enhancing the body’s antitumor immune response.^10^ Clinical evidence indicates that, when combined with chemotherapy or targeted agents, PD-1/PD-L1 inhibitors can improve response rates and survival outcomes.^11^ Oncogenic signaling, especially through PI3K/AKT/mTOR and MAPK/ERK, and IFN-γ-induced JAK/STAT signaling, the main inflammatory pathway leading to inducible PD-L1, constitute the primary pathways regulating PD-L1 in tumors. They form the central regulatory circuitry of PD-L1-mediated immune escape.^12^ The JAK/STAT signaling pathway plays a central role in transmitting signals from cytokine and growth factor receptors to the nucleus. Ligand binding activates Janus kinases (JAKs), which then phosphorylate Signal Transducers and Activators of Transcription (STATs). Once phosphorylated, STATs form dimers, translocate to the nucleus, and regulate the expression of genes involved in cell growth, differentiation, and immune responses. Dysregulation of this pathway is associated with a wide range of diseases, including cancer and autoimmune disorders.^13^^,^^14^ The JAK-STAT pathway regulates both immune defense and cancer progression. While it enhances antitumor immunity via interferon-driven activation of T cells, natural killer (NK) cells, and dendritic cells, its dysregulation, especially sustained STAT3/STAT5 activation, can foster immunosuppression, tumor growth, and therapy resistance.^15^ Targeting the JAK/STAT pathway, particularly STAT3, sensitizes tumors to immunotherapy by simultaneously reversing multiple layers of tumor-driven immunosuppression. Inhibiting this pathway restores dendritic cell maturation and antigen presentation, enhances CD8^+^ T cell and NK cell cytotoxicity, and reduces the number of immunosuppressive cells. It also reduces tumor expression of inhibitory molecules, such as PD-L1, and immunosuppressive cytokines (interleukin [IL]-6, IL-10, transforming growth factor β [TGF-β]). By reshaping both tumor cells and the tumor microenvironment toward a more immunogenic state, JAK/STAT inhibition improves response rates and helps overcome resistance to immune checkpoint inhibitors and other immunotherapies.^16^ Combining JAK/STAT inhibitors with anti-PD-1/PD-L1 therapy offers a promising strategy to overcome immunotherapy.^17^ Beyond breast cancer, extensive preclinical and clinical research across multiple tumor types, including melanoma, lung cancer, pancreatic cancer, colorectal cancer, ovarian cancer, oral squamous cell carcinoma, and hematologic malignancies, has shown that the JAK/STAT pathway is a critical regulator of PD-L1 expression and response to PD-1/PD-L1 blockade. Preclinical work across various solid tumors has shown that inhibiting components of the JAK/STAT pathway, particularly JAK1/2 or STAT3, can enhance the efficacy of PD-1/PD-L1 blockade by reducing PD-L1 expression, restoring antigen presentation, improving dendritic cell activation, and increasing CD8^+^ T cell infiltration.^17^ This review highlights the central role of JAK and STAT signaling in regulating PD-L1 expression, shaping the immune microenvironment in breast cancer, and influencing the response to PD-1- and PD-L1-targeted therapy. Key mechanisms of immune evasion and emerging therapeutic approaches are summarized, providing insights into strategies that may help overcome resistance to immunotherapy.

## Regulation of PD-L1 by JAK/STAT signaling pathway

**Figure 1:**
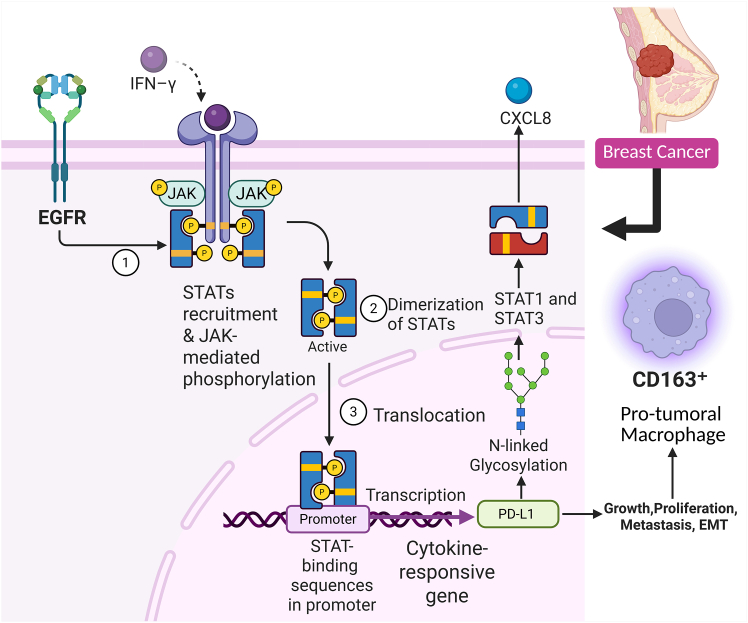
PD-L1 regulation by the JAK/STAT signaling pathway in breast cancer JAK/STAT pathway activation is initiated by IFN-γ binding to its receptor, leading to JAK phosphorylation. This event recruits STAT proteins, which are phosphorylated by JAK kinases. The phosphorylated STATs then dimerize and translocate to the nucleus, binding to cytokine-responsive gene promoter regions, including the PD-L1 promoter, and promoting their expression. Amplification of 9p24.1 in TNBC increases JAK2/STAT3 pathway activation, driving PD-L1 expression and tumor progression. IFN-γ induces PD-L1 transcription via the JAK/STAT signaling pathway, and PD-L1-dependent STAT1 and STAT3 activation play a crucial role in this process. N-linked glycosylation of PD-L1 is essential for STAT activation and its pro-metastatic functions. This axis results in PD-L1 upregulation, facilitating immune evasion, tumor growth, and recruitment of CD163^+^ pro-tumoral macrophages in breast cancer.

**Figure 2:**
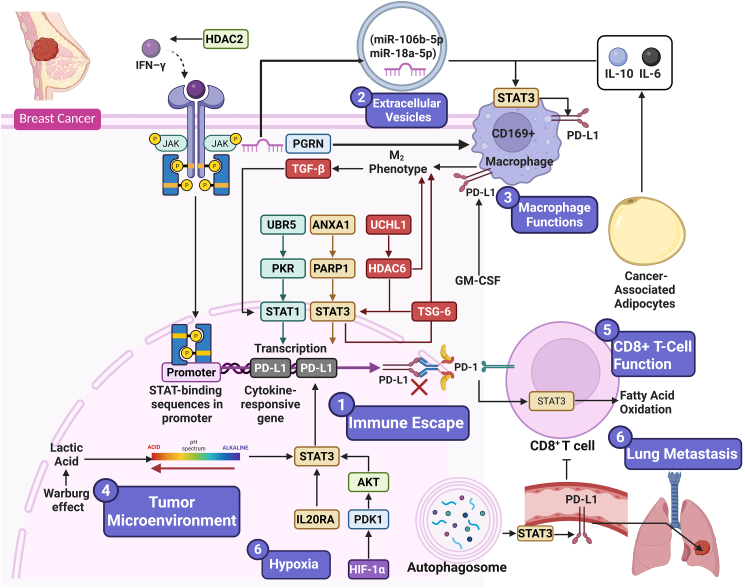
STAT3-Driven Regulation of PD-L1 and Its Role in Breast Cancer Progression This schematic depicts the central role of STAT3 in modulating PD-L1 expression and shaping critical processes involved in breast cancer advancement. STAT3 activation upregulates PD-L1 across tumor cells, immune components, and the broader tumor microenvironment, thereby enabling immune escape through the inhibition of CD8^+^ T cell activity. Interactions among STAT3, tumor-associated macrophages, cancer-associated fibroblasts, and extracellular vesicles further reinforce immunosuppressive signaling and tumor-promoting dynamics. Elucidating these interconnected pathways highlights promising therapeutic opportunities aimed at disrupting the STAT3-PD-L1 axis in breast cancer.

IFN-γ robustly induces PD-L1 expression in breast cancer cell lines. Induction is largely dependent on activation of the JAK/STAT signaling pathway, as evidenced by elevated levels of phosphorylated STAT1 (p-STAT1). In tumor samples from 111 patients, tumors with p-STAT1 positivity exhibited significantly higher PD-L1 and HLA class I expression, further validating an association between JAK/STAT activation and upregulation of immune-related gene signatures. Furthermore, analysis from The Cancer Genome Atlas (TCGA) demonstrated a positive correlation between PD-L1 mRNA expression and IFN-γ signaling and CD8^+^ T cell effector gene signatures.^18^ Amplification of the region of chromosome 9p24.1 encompassing JAK2, PD-L1, and PD-L2 was also shown in TNBC cell lines to be associated with activation of the JAK2/STAT3 pathway and enhanced proliferation. While no direct correlation between basal PD-L1 expression and 9p24.1 CNAs was observed, IFN-γ-induced PD-L1 expression was significantly increased in cell lines that had 9p24.1 gain or amplification. shRNA-mediated knockdown or JAK1/2 inhibition with the drug ruxolitinib inhibited IFN-γ-mediated upregulation of PD-L1 expression through blockade of JAK2/STAT1 signaling.^19^ Basal-type breast cancer cells, in particular the basal B subtype, display significantly greater PD-L1 expression, both at baseline and after interferon γ treatment, than the luminal subtypes. Flow cytometry and cancer cell line encyclopedia (CCLE) mRNA data analysis identified that high PD-L1-expressing cells also exhibit higher expression of invasion, proliferation, and chemoresistance-associated genes, as well as STAT1, and lower IRF2BP2, suggesting a potential regulatory mechanism that increases PD-L1.^20^ STAT1 is a principal mediator of PD-L1 regulation under inflammatory conditions in TNBC cells. Specifically, STAT1 is robustly activated in BT-549 cells stimulated with IFN-γ plus IL-1β, and STAT1 is required for the upregulation of PD-L1 (and PD-L2) expression upon stimulation. This was clearly demonstrated by CRISPR-Cas9 knockout experiments, in which knockout of STAT1, but not p65 (a subunit of nuclear factor κB [NF-κB]), abrogated the cytokine-induced increase in the fraction of cells co-expressing PD-L1 and PD-L2.^21^ Similarly, STAT3 also plays an important role in driving the expression of PD-L1 in breast cancer to promote immune evasion. Strong associations between pSTAT3 and PD-L1 expression were observed in both breast cancer cell lines and patient samples. Pharmacological inhibition of STAT3 and/or STAT3 silencing using siRNA decreased PD-L1 expression. STAT3 suppression in a murine breast cancer model was also shown to inhibit tumor growth and metastasis. Moreover, STAT3 inhibition within the tumor significantly altered the tumor immune microenvironment, enhancing the M1 antitumoral macrophage phenotype and increasing NK cell proliferation. Moreover, in patients, high PD-L1 expression was strongly associated with higher grade, increased proliferation, and a protumoral (CD163^+^) macrophage profile.^20^ In an in-depth mechanistic study, Erlichman et al. demonstrated that PD-L1 mediates pro-metastatic functions in a cell-autonomous manner in breast cancer cells and that this is critically dependent on N-linked glycosylation of PD-L1 and the consequent activation of STAT3 and STAT1. Wild-type PD-L1 overexpression induced robust STAT3 and STAT1 activation in MCF-7 and MDA-MB-231 cells, leading to tumor cell proliferation, invasion, and CXCL8 secretion, an effect largely diminished by S283 mutation or STAT3/STAT1 knockdown with siRNA. Pharmacological inhibitors of N-linked glycosylation, such as kifunensine or swainsonine, led to a lower-molecular-weight PD-L1 protein in cancer cells (consistent with a reduction in glycosylation), without any difference in surface expression of PD-L1, but abrogated STAT3 and STAT1 activation and the cell-autonomous pro-metastatic function of PD-L1. Mutating each of the four putative N-linked glycosylation sites (N35, N192, N200, and N219) one at a time led to loss of pro-metastatic function in all mutants, most strongly in the N219A mutant, which also had the greatest effect on STAT activation and reduction in tumor cell invasion. T cell-independent mouse models of metastasis showed that tumors established with cells expressing the PD-L1 glycosylation mutants (N35A and N219A) were smaller and generated fewer lymph node metastases compared with tumors established with cells expressing wild-type PD-L1. Collectively, these data indicate that the full spectrum of pro-metastatic functions of PD-L1 in breast cancer cells depends on its N-linked glycosylation and the consequent activation of STAT3 and STAT1. That blockade of PD-L1 glycosylation represents a potential new therapeutic strategy in breast cancer.^22^ Loss of HCRP1 results in prolonged EGFR signaling activation, leading to hyperactivation of STAT3. STAT3 hyperactivation results in transcription of a program that upregulates several key oncogenic and immunomodulatory factors, the most notable of which is PD-L1. Loss of HCRP1 *in vitro*, using CRISPR-Cas9 knockout models in ovarian and breast cancer cell lines, resulted in increased proliferation, migration, and a mesenchymal phenotype, characteristic of epithelial-to-mesenchymal transition (EMT). Proteomic analysis confirmed that HCRP1 loss leads to increased membrane retention and phosphorylation of EGFR, thereby enhancing downstream signaling. The upregulation of PD-L1, confirmed at both the RNA and protein levels, was shown to be STAT3 dependent, as STAT3 knockdown rescued PD-L1 expression (Figure 1).^23^ In this section, we discuss in detail how STAT3-mediated regulation of PD-L1 contributes to the cellular mechanisms that drive breast cancer progression (Figure 2).

### Tumor immune evasion

In TNBC, JAK/STAT has also been shown to drive immune escape at the transcriptional level, specifically through the expression of key immunosuppressive factors in this pathway. In TNBC cells, JAK kinases are activated by cytokines and growth factors. JAKs then phosphorylate STAT proteins (STAT3 being the most well studied in TNBC). STAT3 phosphorylation causes it to be translocated to the nucleus, where it drives expression of PD-L1, which binds the PD-1 receptors on CD8^+^ T cells to inactivate and induce apoptosis in those cells.^24^ In TNBC, UBR5 also promotes immune escape by upregulating IFN-γ-induced PD-L1 transcription. This is achieved by upregulating the protein kinase RNA-activated (PKR), which activates the transcription factors STAT1 and IRF1. These factors subsequently bind to the PD-L1 promoter, increasing PD-L1 expression in a PABC-dependent, E3 ligase-independent manner. The overexpressed PD-L1 on tumor cells can suppress T cell activity by interacting with PD-1 receptors on T cells, thereby impairing antitumor immunity and promoting immune escape.^25^ In TNBC, HDAC2 loss blocks IFN-γ-induced PD-L1 expression, thereby failing to suppress immune activity. HDAC2 regulates the phosphorylation of JAK1, JAK2, and STAT1 to allow for nuclear translocation of STAT1, where it directly binds to the PD-L1 promoter. There, it recruits chromatin remodelers, including BRD4, and enhances histone acetylation marks (H3K27ac, H3K9ac) at the PD-L1 promoter, thereby increasing PD-L1 transcription. HDAC2 knockout prevents these events, thereby reducing PD-L1 surface expression. This reduces tumor progression and metastasis and allows for greater lymphocyte infiltration in the tumor microenvironment.^26^ In breast cancer, ANXA1 promotes immune escape by disrupting the interaction between PARP1 and Stat3. PARP1 binds to Stat3 and PARylates it, leading to dephosphorylation and inactivation. ANXA1 sequesters PARP1, preventing it from binding to Stat3. This allows Stat3 to remain phosphorylated and active, thereby increasing PD-L1 expression on tumor cells. The upregulated PD-L1 on tumor cells binds to PD-1 receptors on T cells, inhibiting T cell cytotoxicity and promoting immune escape. This highlights the role of ANXA1 in creating an immunosuppressive tumor microenvironment in breast cancer (Figure 2).^27^

### Extracellular vesicles

Extracellular vesicles (EVs) are crucial in breast cancer because they act like “Trojan Horses,” silently carrying oncogenic cargo (miRNAs, proteins, lipids) between tumor and host cells to reshape the tumor microenvironment and distant organs. Through this vesicle-mediated communication, breast cancer cells promote pre-metastatic niche formation, immune evasion, angiogenesis, metabolic reprogramming, EMT, and drug resistance. At the same time, EVs' natural biocompatibility and targeting ability make them highly promising platforms for precision drug delivery and immunotherapy, positioning them as both key drivers of disease progression and attractive therapeutic tools.^28^ Breast cancer-derived small extracellular vesicles (sEVs) carry microRNAs that are delivered from cancer cells to macrophages, thereby promoting macrophage M2 polarization and tumor progression. Breast cancer-derived sEVs promote tumor progression by providing miR-106b-5p and miR-18a-5p to macrophages. The uptake of these microRNAs by macrophages suppresses the expression of the regulatory proteins PTEN and PIAS3, thereby activating AKT and STAT3. The increased expression of PD-L1 on tumor-associated macrophages (TAMs), driven by this signaling, impairs T cell-mediated immunity, creating an immune-permissive tumor environment that contributes to tumor growth, metastasis, and immune evasion in breast cancer.^29^ Adipose-derived stem cell (ADSC) exosomes become enriched in the deubiquitinating enzyme UCHL1 when exposed to inflammatory stimuli such as IFN-γ and TNF-α. After internalization by TNBC cells, these exosomes elevate UCHL1 intracellular levels, which leads to HDAC6 stabilization and STAT3 pathway activation, resulting in increased PD-L1 expression by tumor cells. Upregulated PD-L1 on tumor cells can bind to inhibitory receptors on T cells, suppressing their cytotoxicity and promoting macrophage polarization toward an M2-like, immunosuppressive phenotype, thereby creating an immune-resistant tumor microenvironment that favors both immune evasion and tumor progression (Figure 2).^30^

### Macrophages function

Breast cancer cells can also drive immunosuppression by upregulating PD-L1 on CD169^+^ macrophages, a process dependent on JAK2/STAT3 signaling. 4T1 breast cancer cells were co-cultured with CD169^+^ macrophages, and the data showed a much higher PD-L1 expression on macrophages than on tumor cells (about 5-fold higher). The data also suggested that this effect depended on JAK2 activation, since phosphorylated JAK2 and STAT3 were upregulated in macrophages during co-culture, and this effect was abrogated with treatment with a JAK2 inhibitor (AZD1480). Depletion of CD169^+^ macrophages suppressed tumor growth and lung metastasis and enhanced CD8^+^ T cell responses in mice.^31^ Further, breast cancer cells can induce cytokines such as IL-6 and IL-10, which are known to activate STAT3 in macrophages. Activated STAT3 in macrophages then induces PD-L1 upregulation on the surface of macrophages, and this results in them becoming immunosuppressive. Upregulated PD-L1 on macrophages then binds to PD-1 on T cells, inhibiting their activation and proliferation. STAT3, by inducing PD-L1 expression, helps the tumor evade immune surveillance by limiting effective T cell responses and creates a feedback loop that further promotes immunosuppression in the tumor microenvironment [32]. In the breast cancer microenvironment, cancer-associated adipocytes (CAAs) contribute to immunosuppression by secreting high levels of IL-6. This IL-6 can then activate STAT3 signaling and promote macrophage polarization toward an M2-like phenotype. M2 macrophages are characterized by high expression of immunosuppressive molecules, including PD-L1, which can inhibit antitumor immune responses and promote tumor growth.^32^ Additionally, CAAs not only enhance the invasiveness and proliferation of breast cancer cells but also intensify immunosuppression by increasing the recruitment and accumulation of M2 macrophages within the tumor microenvironment.^33^ An interesting study by Gómez et al. (2020) has recently highlighted that breast cancer-associated macrophages actively reprogram tumor cell metabolism to favor cancer progression, particularly in macrophages with an anti-inflammatory (M2-like) phenotype. These macrophages secrete TGF-β, which inhibits the transcription factor STAT1 in breast cancer cells; this, in turn, contributes to a STAT1-dependent downregulation of the enzyme succinate dehydrogenase (SDH). SDH is required for both the tricarboxylic acid cycle and oxidative phosphorylation, so its downregulation is associated with accumulation of succinate, stabilization of HIF1α, metabolic reprogramming of the tumor cells toward glycolysis (the Warburg effect), enhanced angiogenesis, and upregulation of the immunosuppressive protein PD-L1.^34^ Lastly, progranulin (PGRN) has also been implicated in immune escape of breast cancer. PGRN induced TAMs to acquire an M2 phenotype and upregulated the expression of PD-L1 on TAMs by activating the STAT3 signaling pathway. *In vitro* and *in vivo*, treatment with PGRN increased M2 markers and PD-L1 expression on macrophages, spatially excluding CD8^+^ T cells from the tumor parenchyma and suppressing their activation and proliferation. The role of PD-1/PD-L1 was confirmed using blocking antibodies, showing that neutralizing PD-1 and PD-L1 rescued the suppression of CD8^+^ T cell function (Figure 2).^35^

### Tumor microenvironment

In MDA-MB-231 breast cancer cells, the acidic pH of the TME contributes to increased PD-L1 expression, predominantly through the activation of the STAT3 signaling pathway. Acidosis, resulting from the excessive production of lactic acid by cancer cells via the Warburg effect, lowers extracellular pH. This reduction in pH induces the phosphorylation of STAT3 at tyrosine 705, activating it. Activated STAT3 then translocates to the nucleus and binds to the promoter regions of target genes, including PD-L1, resulting in increased PD-L1 expression. The upregulation of PD-L1 on the surface of cancer cells contributes to immune evasion by inhibiting T cell function. Significantly, interventions that neutralize the acidic pH or directly inhibit STAT3 activity can reduce PD-L1 expression.^36^ In human breast cancer, inflammatory signals from activated lymphocytes induce PD-L1 expression on TAMs. In particular, granulocyte-macrophage colony-stimulating factor (GM-CSF) from activated lymphocytes, together with IFN-γ, activates the STAT3 signaling pathway in macrophages, which causes strong upregulation of PD-L1 on the macrophage surface and results in an immunosuppressive microenvironment that hinders lymphocyte activation and facilitates tumor immune escape.^37^ SiRNA-mediated knockdown of TSG-6 expression in human and canine breast cancer cells impaired their proliferative, migratory, and invasive potential, accompanied by changes in cell cycle. TSG-6 downregulation also reduced the expression of key signaling molecules, including NF-κB, STAT3, and Sox2, as well as stem cell marker CD44 and immune checkpoint protein PD-L1. Co-culture studies further revealed that TSG-6 knockdown in tumor cells could reprogram macrophages from an immunosuppressive M2 phenotype to a pro-inflammatory M1 phenotype. It also increased the activation and cytotoxicity of CD3^+^/CD8α^+^ T cells with upregulation of PD-1 and downregulation of CTLA-4 (Figure 2).^38^

### CD8^+^ cells

Metabolic reprogramming in breast cancer is the capacity of the cancer cells to rewire the cellular metabolism. This includes alterations in energy-yielding pathways to support cancer cells. These changes can include increased glucose uptake, increased glycolysis, alterations in lipid metabolism, and alterations in amino acid metabolism. These changes contribute to the breast cancer cells' growth, survival, and resistance to therapy.^39^ Accumulating evidence has suggested that breast tumor progression in obesity is significantly associated with a severe defect in the effector functions of tumor-infiltrating CD8^+^ T cells. The study found that obese mice on a high-fat diet developed significantly larger tumors with earlier onset and more metastasis. In addition, there was also lower expression of significant cytolytic markers, including IFN-γ, granzyme B, and CD107a, in tumor-infiltrating CD8^+^ T effector cells. Further mechanistic studies demonstrated that STAT3 activation in CD8^+^ T cells upregulated fatty acid oxidation (FAO) by inducing the expression of the rate-limiting enzyme CPT1B. At the same time, STAT3 suppressed glycolysis. Moreover, both PD-1 engagement and leptin signaling further enhanced STAT3 activation, thus amplifying the effects of FAO upregulation and glycolysis suppression. The findings provide mechanistic evidence of how obesity impairs the antitumor activity of CD8^+^ T cells and creates an immunosuppressive microenvironment. Mechanistically, blocking T cell STAT3 or pharmacologically inhibiting FAO using inhibitors such as etomoxir and perhexiline to restore glycolytic metabolism in T cells and reactivate T cell effector function reduced tumor growth and metastasis.^40^ In human breast cancer, TAMs have been shown to acquire increased PD-L1 expression through inflammatory signals from activated lymphocytes. Conditioned media from activated lymphocytes, but not from resting lymphocytes or breast cancer cells, significantly induced overexpression of PD-L1 *in vitro* by human monocyte-derived macrophages. It was identified that GM-CSF secreted by activated lymphocytes, in the presence of IFN-γ, activated the STAT3 signaling pathway, further upregulating PD-L1 expression on macrophages. Furthermore, this increased PD-L1 expression was functionally relevant, suppressing lymphocyte activation, and this suppression could be blocked by PD-1 blockade. Analysis of clinical specimens as well as TCGA data showed a positive correlation of PD-L1 expression with both macrophage markers and CD8 T cell-associated genes, emphasizing the clinical relevance of the inflammatory microenvironment in regulating immune checkpoint expression on TAMs (Figure 2) [33].

### Lung metastasis

Autophagosomes released by breast cancer cells (TRAPs) can actively induce lung metastasis by promoting immunosuppressive pre-metastatic niche formation. TRAPs enter the lung and drive upregulation of PD-L1 on pulmonary vascular endothelial cells through HMGB1-dependent TLR4-MyD88-p38/STAT3 signaling. PD-L1 on endothelial cells suppresses CD4^+^ and CD8^+^ T cell activation and proliferation, thereby repressing antitumor immune response. Restoration of T cell function by anti-PD-L1 antibody therapy can also effectively reduce lung metastasis. This highlights the importance of early immunotherapeutic strategies to counteract TRAP-induced immunosuppression in breast cancer.^41^ IL20RA expression in breast cancer cells has also been shown to increase cancer stem cell-like properties, including the side population, sphere-forming capacity, and ALDH activity, and to mediate chemoresistance. Mechanistically, IL20RA overexpression activated JAK1-STAT3, which in turn induced expression of stemness transcription factors SOX2, OCT4, and Nanog, thereby driving tumor initiation and lung metastasis *in vivo*. Furthermore, IL20RA signaling in tumor cells was found to contribute to an immunosuppressive tumor microenvironment by upregulating PD-L1, reducing infiltration of cytotoxic immune cells, including CD8^+^ T cells and NK cells, and enhancing infiltration of myeloid-derived suppressor cells (MDSCs). It was also shown that a novel therapeutic approach combining IL20RA-mediated targeted delivery of a STAT3 inhibitor with anti-PD-L1 immunotherapy and chemotherapy significantly enhanced therapeutic efficacy in mice (Figure 2).^42^

### Hypoxia

Under hypoxic conditions, HIF-1α is stabilized and binds directly to the FTO promoter, leading to increased FTO expression. Elevated FTO then demethylates m6A modifications on PDK1 mRNA, which prevents YTHDF3-mediated degradation and enhances its stability. Increased PDK1 levels activate the AKT pathway, leading to STAT3 phosphorylation and activation, which in turn drives PD-L1 transcription. This cascade not only promotes tumor immune evasion but is also supported by *in vivo* findings showing that inhibition of FTO and PDK1 reduced tumor growth and enhanced cytotoxic T cell activity. Moreover, clinical data indicate that high levels of FTO and PDK1 correlate with poorer prognosis in breast cancer patients, underscoring the potential of targeting this pathway to improve therapeutic outcomes (Figure 2).^43^

### BRCA1/2 and effect on the PD-L1 and JAK/STAT interplay

Gao et al. showed that BRCA1 overexpression leads to constitutive activation of JAK/STAT3 signaling in prostate cancer cells and that blocking STAT3 induces apoptosis. This is direct evidence that BRCA1 can modulate JAK/STAT activity.^44^ Multiple mechanistic papers show that BRCA1 acts as a co-activator of interferon signaling: BRCA1 is required for upregulation of STAT1, STAT2, and type I IFNs in response to IFN-γ, effectively priming cells for IFN/JAK/STAT responses.^45^ Ezell et al. (“Akt1, EMSY, BRCA2, and type I IFN signaling”) showed that BRCA2 participates in the regulation of type I IFN responses, which are classically mediated by the JAK/STAT pathway.^46^ BRCA2 loss or abrogation triggers innate immune activation via cGAS/STING and type I interferon signaling, with downstream dependence on STAT1 for full induction of interferon-stimulated genes.^47^ A systems biology analysis of BRCA1/2-mutant vs. wild-type breast cancers identified co-expression modules specific to BRCA1/2 carriers, enriched for pathways involved in immune response and JAK/STAT signaling, among others.^48^ In addition, BRCA2 mutations are associated with upregulated IL-6-related signaling, which is a canonical activator of JAK/STAT3; the pathway patterns differ between BRCA1- and BRCA2-driven tumors.^48^ BRCA1 and BRCA2 mutations differentially affect the tumor microenvironment and response to immune checkpoint blockade across several cancers. BRCA2-mutant tumors tended to have higher tumor mutational burden (TMB) and better responses compared with BRCA1-mutant tumors.^49^ Importantly, reviews of PARP inhibition and antitumor immunity note that BRCA1/2 and other DNA damage response (DDR) defects increase baseline pro-inflammatory signaling, particularly type I IFN and JAK/STAT and that this is associated with enhanced sensitivity to immune checkpoint inhibitors and synergy with PARP inhibitors.^50^

### Differences in JAK/STAT-PD-L1 regulation among different breast cancer subtypes

Breast cancer is a biologically heterogeneous disease, and current evidence indicates that JAK/STAT-mediated regulation of PD-L1 is modulated by intrinsic subtype. Basal-type, particularly basal B and TNBC models, consistently show higher basal and inducible PD-L1 expression compared with luminal subtypes, in association with elevated STAT1 activity and pro-invasive, chemoresistant gene signatures. Flow cytometry and CCLE mRNA analyses have demonstrated that basal-type cells not only express more PD-L1 at baseline but also exhibit a stronger PD-L1 induction after IFN-γ stimulation than luminal counterparts, underscoring the dominant role of JAK/STAT, especially STAT1, in driving PD-L1 in these aggressive subtypes.^20^

In hormone receptor-positive/HER2-negative (luminal) breast cancer, JAK/STAT-PD-L1 regulation appears more nuanced. In HR^+^ cell lines (such as MCF-7 and T47D), exposure to a “luminal-like” microenvironmental cocktail of estrogen, TNF-α, and EGF increases PD-L1 expression together with enrichment of cancer stem-cell and EMT-like features.^22^ Pharmacologic inhibition of STAT3 with Stattic reduces PD-L1 levels and STAT3 phosphorylation in these models, indicating that even in HR^+^/HER2^-^ tumors, PD-L1 is at least partly under STAT3 control. Interestingly, clinical data in luminal A breast cancer suggest that higher STAT3/p65 phosphorylation correlates with *lower* CSC-related gene signatures and more favorable outcomes, implying that the consequences of modulating STAT3 may differ between luminal and basal/TNBC contexts and should be interpreted in a subtype-specific manner.^51^

In HER2-positive breast cancer, JAK/STAT-dependent PD-L1 regulation is closely linked to trastuzumab response. In HER2^+^ SK-BR-3 cells, combining the selective STAT3 inhibitor FLLL32 with Herceptin more effectively reduces STAT3 phosphorylation, vascular endothelial growth factor (VEGF), and PD-L1 expression than either agent alone and concurrently decreases multiple immune checkpoints (PD-1, CTLA-4, TIM-3) on lymphocytes while enhancing IFN-γ/IL-18 production and CD8^+^ T cell cytotoxicity.^52^ These findings indicate that STAT3-driven PD-L1 upregulation contributes to immune escape and trastuzumab resistance in HER2^+^ tumors and that simultaneous blockade of HER2 and STAT3 can resensitize tumors by lowering PD-L1 and restoring antitumor immunity.^53^ In addition, apigenin was shown to inhibit IFN-γ-induced PD-L1 upregulation through STAT1 inhibition in HER2^+^ SK-BR-3 cells, further supporting the notion that both STAT1 and STAT3 participate in fine-tuning PD-L1 expression in HER2-driven disease.^54^

## Regulation of JAK/STAT signaling by PD-L1

Blocking PD-L1 potently suppressed STAT3 activation, which is crucial for M2 polarization. When treated with IL-13, STAT3 became phosphorylated and translocated into the nucleus. There, it activated genes that regulate the M2 phenotype and tumor-promoting functions. In the presence of PD-L1 blockade, STAT3 phosphorylation is significantly impaired, thus failing to translocate into the nucleus. Reduced transcription of target genes downstream of STAT3 leads to macrophage reprogramming away from the protumorigenic M2 state.^55^ In TNBC, analysis of tumor tissues derived from patients with aggressive disease revealed that they released PD-L1-enriched MPs, which strongly correlated with tumor burden and lung metastases. Consistent with a role for PD-L1 in immune-mediated progression, deletion of PD-L1 from tumor cells in immunocompetent mouse models significantly reduced primary tumor burden and metastatic lesion numbers. In the same models, treatment with PD-L1-bearing MPs suppressed CD8^+^ T cell function, correlating with decreased production of cytotoxic molecules, including IFN-γ, perforin, and granzyme B, and resulting in diminished antitumor immunity. In addition to suppressing T cell immunity, PD-L1-bearing MPs in these models polarized macrophages toward an immunosuppressive M2 phenotype, as evidenced by increased expression of CD206 and IL-10. Mechanistically, the M2 shift was driven by activation of the cGAS/STING/TBK1/STAT6 axis and inhibition of AKT/mTOR. Finally, established clinical interventions, including chemotherapy and radiotherapy, were shown to increase the release of PD-L1-laden MPs, which exacerbated immunosuppression and promoted TNBC progression.^56^

## JAK/STAT and PD-L1 Interplay in response to anti-PD-1

**Figure 3:**
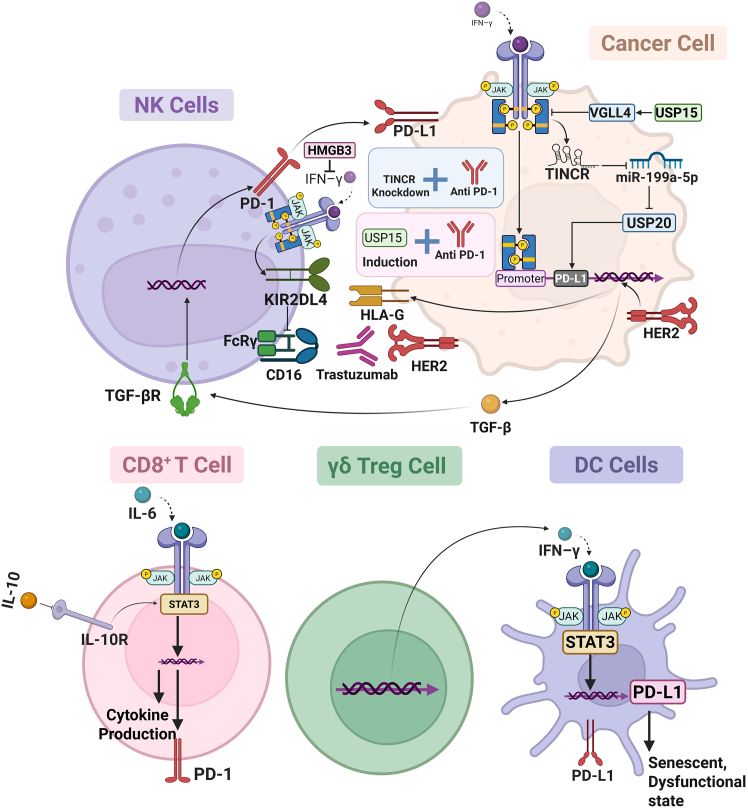
JAK/STAT and PD-L1 axis in the tumor immune microenvironment of anti-PD-1 therapy A schematic overview of interactions and signaling events between tumor cells and immune cells, including NK cells, CD8^+^ T cells, γδ Treg cells, and dendritic cells, in the context of immune checkpoint blockade inhibitors of PD-1/PD-L1 for breast cancer. Tumor cell-intrinsic factors encompass IFN-γ-driven JAK/STAT signaling pathways that increase PD-L1 expression and promote adaptive resistance to anti-PD-1 immunotherapy. For example, HMGB3 overexpression attenuates STAT1 activation and promotes STAT3 signaling, thereby contributing to adaptive resistance to anti-PD-1 therapy by modulating IFN-γ-induced cytotoxicity and ferroptosis inhibition. TINCR also mediates immune evasion in tumor cells by promoting PD-L1 expression via dual nuclear and cytoplasmic mechanisms, including miR-199a-5p suppression and USP20 stabilization. USP15 stabilizes VGLL4 and inhibits STAT3 phosphorylation, thereby decreasing PD-L1 transcription. Immune cell-intrinsic factors also modulate responses to anti-PD-L1 therapy. In CD8^+^ T cells, IL-6R-driven STAT3 activation skews them toward less differentiated phenotypes with impaired effector functions. In another CD8^+^ T cell pathway, IL-10 receptor signaling also supports maintenance of a memory-like T cell subset. NK cells provide a mechanism of trastuzumab efficacy through ADCC involving CD16, and NK cell activity is inhibited through HLA-G on tumor cells binding to KIR2DL4 receptors on NK cells, which JAK/STAT also controls. γδ Treg cells cause DC senescence and dysfunction through STAT3-dependent PD-L1 upregulation, which causes a reduction in antigen presentation.

The JAK/STAT pathway transduces the cellular response to IFN-γ that is produced following immune activation. As such, this pathway mediates IFN-γ-induced PD-L1 expression on tumor cells. In the context of anti-PD-1 immunotherapy, the JAK/STAT pathway serves as a compensatory negative feedback mechanism. While the blockade of PD-1 restores T cell effector function, IFN-γ-induced JAK/STAT signaling can also drive PD-L1 expression, contributing to adaptive resistance. The opposing actions of this pathway may therefore dampen the therapeutic response. As such, inhibition of JAK/STAT signaling may be required to increase the efficacy of anti-PD-1 immunotherapy and reverse resistance.^17^ Similarly, high STAT4 expression (notably in triple-negative tumors), which is associated with better patient survival, is mainly due to its expression in tumor-infiltrating T cells. While STAT4 overexpression *in vitro* enhanced tumor cell proliferation, migration, and invasion, it inhibited tumor growth *in vivo* by enhancing the antitumor immune response. Mechanistically, STAT4 was shown to work in conjunction with STAT3 to transactivate the interleukin receptors IL12RB1 and IL12RB2. This was the result of a positive feedback loop in which IL-12R/JAK2/STAT3/STAT4 signaling eventually led to upregulation of PD-L1 expression. The STAT4-related pathway score (Srps) was highly associated with T cell expansion and represents a viable biomarker for predicting response to anti-PD1 immunotherapy, with significant potential to guide patient stratification and personalized immunotherapy in breast cancer.^57^ Activation of FGFR2 signaling by a S252W mutation in the mammary gland causes the development of TNBC tumors. The S252W mutation-induced breast tumors are characterized by increased mammary branch morphogenesis and expansion of mammary stem-like cells. Mechanistically, FGFR2 activation leads to activation of the STAT3 and ERK pathways, resulting in EMT and increased PD-L1 expression, thereby contributing to an immunosuppressive microenvironment. FGFR2 also represses BRCA1 transcription by downregulating the transcription factor YY1 via the FRS2α/STAT3/MAPK signaling cascade, leading to accelerated tumorigenesis, which is further exacerbated by BRCA1 deficiency. Inhibition of FGFR2 signaling, alone or in combination with immune checkpoint blockade using a PD-1/PD-L1 antibody, efficiently inhibits FGFR2-induced tumor growth, as shown in a fast tumor slice culture platform that mimics the drug combination treatment strategy for FGFR2-driven breast cancer.^58^ PARP inhibitors are single-agent drugs that target homologous recombination deficiency, primarily through synthetic lethality in breast and other tumors with BRCA1/2 mutations. The review by Zou et al. nicely summarizes the primary mechanisms of tumor resistance to PARP inhibition and the emerging approaches to combination therapy, especially with immune checkpoint blockade, to resensitize tumors. This is closely related to our study, as the DNA damage caused by PARPi can induce inflammatory and cytokine programs (including JAK/STAT-associated programs) that are both responsible for immunogenicity and adaptive immune escape. The study bridged the biology of PARPi resistance with immune modulation, supporting our focus on JAK/STAT alterations as drivers of response to immunotherapy and potential biomarkers for rational combination approaches in breast cancer.^59^ In this section, we review the mechanisms that contribute to anti-PD-L1 therapy efficacy in breast cancer (Figure 3).

### Cancer cells-mediated mechanisms

HMGB3 overexpression in TNBC also contributes to resistance to anti-PD-1 immunotherapy by suppressing IFN-γ-driven antitumor activity. It was found that mice with HMGB3-deficient tumors showed smaller tumors and prolonged survival when treated with an anti-PD-1 antibody *in vivo*. Similarly, higher HMGB3 levels were associated with poorer overall survival and progression-free survival in clinical data from patients with TNBC. Mechanistically, HMGB3 suppresses IFN-γ-induced cytotoxicity by inhibiting STAT1 phosphorylation and IRF1 expression, and by inducing STAT3, thereby impairing the tumor-killing immune response. Moreover, HMGB3 attenuated IFN-γ-induced ferroptosis by upregulating the ferroptosis-inhibitory proteins SLC7A11, GPX4, and SLC3A2, which further shields TNBC cells against ferroptosis-mediated cell death.^60^ LncRNAs can act as molecular “sponges” by binding and sequestering specific miRNAs, thereby preventing those miRNAs from repressing their target mRNAs and ultimately increasing the expression of the miRNA-regulated genes.^61^ Similarly, the long non-coding RNA TINCR contributes to breast cancer progression and immune evasion. TINCR increases PD-L1 protein expression through two complementary mechanisms. In the nucleus, it recruits DNMT1 to methylate the miR-199a-5p promoter, thereby suppressing its transcription. In the cytoplasm, TINCR functions as a competing endogenous RNA, sponging miR-199a-5p and stabilizing USP20 mRNA. Increased USP20 then reduces the ubiquitination and degradation of PD-L1, resulting in higher PD-L1 protein levels. IFN-γ further enhances TINCR transcription via STAT1 activation, leading to upregulation of USP20 and PD-L1 and ultimately weakening the therapeutic efficacy of PD-L1 inhibitors. *In vivo* studies showed that TINCR knockdown markedly reduced tumor growth and improved the response to PD-L1 blockade, identifying TINCR as a promising therapeutic target in breast cancer immunotherapy.^62^ Similarly, USP15 can also enhance the effectiveness of tumor immunotherapy in TNBC by stabilizing VGLL4 through deubiquitination of K48-linked ubiquitin chains. Stabilization of VGLL4 can suppress STAT3 phosphorylation, thereby inhibiting PD-L1 transcription. At the same time, low USP15 expression in patients was associated with higher PD-L1 expression, lower CD8^+^ T cell infiltration, and poor prognosis. In contrast, high USP15 expression was associated with better immune cell infiltration and better immunotherapy outcomes. In addition, in addition to membrane-bound PD-L1, nuclear PD-L1 also promotes tumor growth and genome stability. In summary, the above studies showed that the USP15-VGLL4-STAT3 axis is an essential regulator of PD-L1, suggesting that targeting this axis could improve the efficacy of PD-1/PD-L1 blockade therapy in TNBC.^63^

### CD8^+^ cells-mediated mechanisms

CD8^+^ T cell-intrinsic IL-6 signaling can confer resistance to anti-PD-L1 immunotherapy by directly inhibiting cytotoxic T lymphocyte (CTL) effector differentiation and function. IL-6 signaling via the IL-6 receptor on CD8^+^ T cells leads to STAT3 activation and downstream transcription factor expression, including BATF. This signaling axis represses the expression of effector molecules, including IFN-γ, TNF, and granzyme B; downregulates co-stimulatory receptors; and upregulates inhibitory molecules, resulting in a more naive or memory-like CTL phenotype with impaired proliferation and cytotoxic activity in response to potent T cell receptor and co-stimulatory signals. In the tumor microenvironment, this IL-6-induced reprogramming of CTLs limits the ability of anti-PD-L1 therapy to promote a robust and polyfunctional CTL response, leading to impaired tumor regression and therapeutic resistance.^64^ IL-10 receptor signaling is required to maintain a functional memory-like CD8^+^ T cell population with intermediate PD-1 and TCF-1 expression, which is necessary for long-term antitumor immunity. Blockade of IL-10R signaling or genetic deletion of IL-10RB or STAT3 causes a dramatic shift toward the accumulation of terminally exhausted PD-1-CD8^+^ T cells, which secrete fewer cytokines, exhibit decreased degranulation, and have reduced proliferative capacity. In line with this, chromatin accessibility assays show that IL-10R blockade results in a shift in the epigenetic landscape, with decreased accessibility of critical loci associated with effector and memory function and disruption of cooperative NFAT and AP-1 transcription factor activity. Consistent with IL-10’s role in tumor immunity, increased IL-10 expression correlates with improved outcomes in breast cancer patients. Exhaustion is a common feature of CD8^+^ T cells across other tumor types, and these studies have been validated in models of colorectal cancer and melanoma, suggesting a more global role of the IL-10R-STAT3 axis in mediating the fine-tuning of T cell activation and exhaustion.^65^

### NK cell-mediated mechanisms

NK cells are key effectors in trastuzumab-induced antitumor immunity in HER2-positive breast cancer. NK cells mediate antibody-dependent cellular cytotoxicity (ADCC) against tumor cells through activating receptors, such as CD16. In contrast, binding of the nonclassical HLA-G antigen expressed on tumor cells to the NK cell receptor KIR2DL4 inhibits NK cell responses. In the absence of HLA-G, KIR2DL4 can promote NK cell activation via FcRγ-dependent signaling, thereby increasing IFN-γ production and cytotoxicity. Moreover, IFN-γ increases KIR2DL4 expression via the JAK2/STAT1 signaling pathway, creating a positive feedback loop that further activates NK cells. However, when KIR2DL4 binds HLA-G, NK cell activation is impaired, leading to trastuzumab resistance. In addition, TGF-β and IFN-γ regulate the expression of HLA-G, KIR2DL4, and immune checkpoints PD-1/PD-L1 to suppress NK cell responses. Critically, inhibition of HLA-G/KIR2DL4 interaction restores NK cell cytotoxicity and augments *in vivo* efficacy of trastuzumab, pointing to an actionable mechanism to reverse resistance in HER2-positive breast cancer.^53^

### Treg cells-mediated mechanisms

Breast cancer-derived γδ Treg cells interact with dendritic cells (DCs) and push them toward a senescent and dysfunctional state in a STAT3/PD-L1 axis-dependent manner. γδ Treg cells upon contact with DCs were able to induce STAT3 phosphorylation in DCs, leading to activation of multiple intracellular signaling cascades and transcriptional upregulation of PD-L1. Senescent DCs with increased PD-L1 expression are unable to mature and fully present antigens and have been shown to have impaired antigen-presenting cell function. Senescent DCs exhibit reduced expression of key costimulatory and maturation molecules, including CD83, CD80, CD86, and HLA-DR, and decreased capacity to produce proinflammatory cytokines. The low antigen-presenting capacity in these senescent DCs impairs activation and proliferation of tumor-specific effector T cells. Also, it increases the differentiation of naive T cells to other regulatory T cells. Therefore, the STAT3/PD-L1 axis in γδ Treg cells promotes senescence and dysfunction in DCs.^66^

## Targeting STATs can be used to boost anti-PD-1 therapy

Combined STAT1 and STAT3 inhibition synergistically reduced PD-L1 expression in human breast cancer cells. This phenomenon occurs by blocking the major signaling pathway involved in its transcription. STAT1 and STAT3 are persistently activated in these cancer cells through phosphorylation. The activated forms of these proteins were shown to dimerize in the cytosol as a STAT1/STAT3 heterodimer before translocating into the nucleus and binding to specific DNA response elements in the PD-L1 promoter to drive its persistent expression. Inhibiting STAT1 or STAT3 alone only partially represses PD-L1 expression. Dual inhibition of STAT1 and STAT3, on the other hand, results in complete repression of PD-L1. This is because this regimen blocks the nuclear translocation of the pSTAT1-pSTAT3 dimer.^67^ Similarly, combinatorial treatment with the STAT3-selective inhibitor FLLL32 and Herceptin (a monoclonal antibody against the HER2 receptor) increased apoptosis in HER2-positive SK-BR-3 breast cancer cells compared with monotherapies. Combination treatment also reduced STAT3 phosphorylation, VEGF, and PD-L1 expression. The treatment reduced the levels of immune inhibitory checkpoints (PD-1, CTLA-4, TIM-3) on lymphocytes. It also led to a shift in the immune response toward a protective Th1 profile, as evidenced by increased IFN-γ and IL-18 production and enhanced CD8^+^ T cell cytotoxicity. These data indicate that inhibition of STAT3 may improve the efficacy of Herceptin and could be a strategy to overcome resistance to HER2-targeted therapy in breast cancer.^52^ Loss of Stat3 signaling in cancer cells with Stat3 addiction leads to cellular senescence and the onset of senescence-associated secretory phenotype (SASP). The SASP includes induction of type I interferon-related cytokines and chemokines such as CXCL10, CCL2, CCL5, and IL-15. This results not only in the inhibition of tumor cell proliferation and migration, but also in the activation of the immune response and enhanced functions of CD4^+^ T cells, NK cells, and CD8^+^ T cells. Furthermore, combined with anti-PD-1 treatment, SASP-based immunotherapy targeting Stat3-silenced cells remarkably improved tumor control and survival in preclinical settings, and the cGAS/STING pathway was a key mediator of SASP-induced immune-stimulatory effects.^68^ In this section, we outline the cellular mechanisms by which inhibiting each component of the JAK/STAT pathway can enhance the efficacy of anti-PD-L1 therapy.

### STAT1

In TNBC, STAT1 inhibition enhances the effectiveness of immune checkpoint blockade by reshaping the tumor microenvironment in favor of immune activation. In MCT-primed tumors, STAT1 activity in a subset of immature myeloid cells drives the expression of immunosuppressive factors such as PD-L1, which suppresses T cell function despite inflammatory signals. Blocking STAT1 reduces PD-L1 expression in these myeloid cells and shifts the immune landscape toward a more pro-inflammatory state. This change limits the recruitment of additional suppressive populations, including CXCL16-high cells, and promotes robust T cell infiltration and activation. Combined treatment with STAT1 inhibition and anti-PD-1 therapy produces a synergistic antitumor response, resulting in greater tumor control and prolonged progression-free survival. These findings indicate that targeting STAT1 may help overcome immunotherapy resistance in TNBC.^69^

### STAT3

Breast cancer-associated fibroblasts produce increased levels of IL-6 compared with normal fibroblasts. Furthermore, an elevated IL-6 environment is a potent inducer of PD-L1 expression in TNBC cell lines via STAT3 and AKT activation. High IL-6 conditioned media is also able to induce resistance to doxorubicin-induced apoptosis and diminish the cytotoxic activity of FRα CAR T cells in 2D and 3D models. Silencing of the IL-6 receptor or inhibiting STAT3 or AKT signaling pathways significantly reduces PD-L1 upregulation and restores sensitivity to chemotherapy and CAR T cell-mediated killing.^70^ In TNBC cells, doxorubicin-induced DNA damage leads to STING-dependent NF-κB activation and subsequent IL-6 production, which, in turn, drives autocrine STAT3 phosphorylation, promoting cell survival and immune evasion through increased PD-L1 expression. STING knockdown not only sensitizes TNBC cells to genotoxic stress, reducing their clonogenicity and spheroid-forming ability, but also decreases PD-L1 expression, thereby enhancing immune cell-mediated cytotoxicity. Furthermore, the combination of doxorubicin and a STAT3 inhibitor shows synergistic apoptosis-promoting effects. Clinical correlations indicate that higher STING expression in TNBC is associated with increased IL-6 and PD-L1 levels, which correlate with reduced survival in patients treated with chemotherapy.^71^ Breast cancer cells can evade immune detection via a newly identified SOX9-to-B7x signaling axis. In dedifferentiated, stem-like tumor populations, the transcription factor SOX9 is upregulated and increases expression of the immune checkpoint molecule B7x by binding directly to the VTCN1 gene and activating STAT3 signaling. Elevated B7x suppresses T cell infiltration and cytotoxic activity, particularly in early-stage lesions such as ductal carcinoma *in situ*, which facilitates progression to invasive disease. Inhibition of B7x restores T cell-mediated immunity, reduces tumor growth, and improves the response to anti-PD-L1 therapy. These findings identify the SOX9-to-B7x pathway as a promising therapeutic target in basal-like breast cancers with poor immune infiltration.^72^ Regorafenib also mediates immunogenic cell death (ICD) in TNBC through increased exposure of damage-associated molecular patterns (DAMPs), such as calreticulin, HMGB1, and ATP, likely due to inhibition of STAT3 signaling. Reduced viability and increased apoptosis of 4T1 TNBC cells *in vitro* were associated with significant upregulation of all three DAMPs. *In vivo*, regorafenib treatment significantly suppressed tumor growth and metastasis and was associated with increased recruitment of both CD4^+^ and CD8^+^ T cells. Dual therapy with anti-mPD-1 antibodies also expanded populations of activated CD8^+^ T cells in the spleen with a CD44ˆhighCD62Lˆlow phenotype; however, the combination did not exhibit any synergistic antitumor activity compared with each agent alone. Pharmacological inhibition of the Jak2 and STAT3 pathways further mitigated immunosuppression by reducing the prevalence of regulatory T cells and driving dendritic cell maturation, which are critical for antigen presentation and activation of cytotoxic T cells.^73^ The AURKA inhibitor MLN8237 increases PD-L1 expression in breast and colon cancer cells by activating STAT3, thereby reducing CD3^+^ and CD8^+^ T cell infiltration and weakening antitumor immunity. AURKA knockdown produces similar effects, confirming its role in PD-L1 regulation. *In vivo*, MLN8237 reduces T cell infiltration into tumors, but combining it with anti-PD-L1 therapy reverses this immunosuppression and significantly improves tumor control. These results explain the limited clinical efficacy of MLN8237 as a single agent and support combined treatment with immune checkpoint inhibitors to counteract MLN8237-induced immune escape.^74^

## Therapeutic targeting of PD-L1/STAT pathway

**Figure 4:**
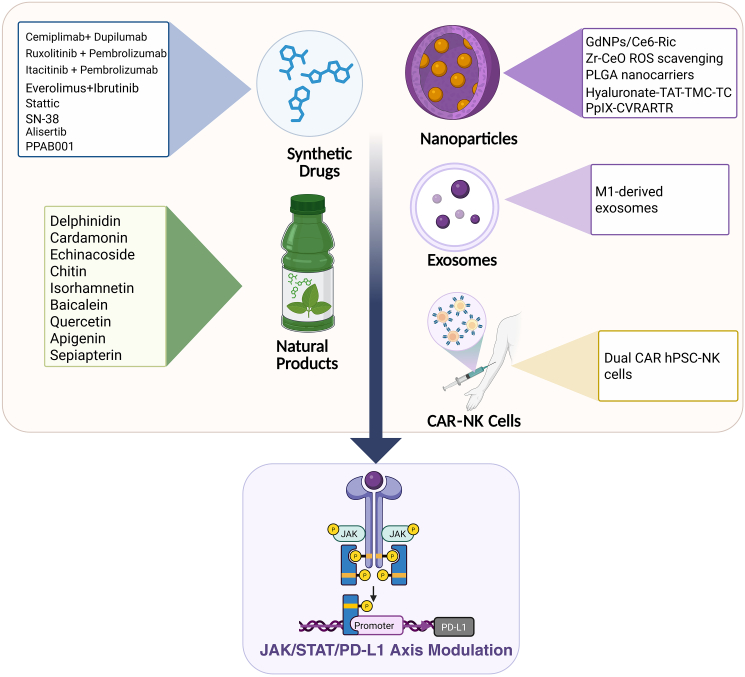
Therapeutic strategies targeting the PD-L1/STAT pathway in cancer immunotherapy This schematic illustration highlights multiple innovative approaches developed to disrupt the JAK/STAT signaling axis, critical in regulating PD-L1 expression and immune evasion by cancer cells. The strategies include the application of synthetic drugs (e.g., Everolimus,^75^ Ibrutinib,^75^ AZD1775 [79], Stattic,^51^ SN-38,^76^ Alisertib,^77^ MEKi,^78^ and PPAB001^79^), nanoparticle-based delivery systems (e.g., GdNPs/Ce6-Ric,^80^ ROS-scavenging nanozymes,^81^ PLGA nanocarriers,^82^ Hyaluronate-TAT-TMC-TC,^83^ and photodynamic immunostimulators^84^), bioengineered exosomes targeting immunosuppressive pathways,^85^ CAR-engineered NK cells designed to enhance antitumor activity,^86^ and natural products (e.g., cardamonin,^87^ delphinidin,^88^ echinacoside,^89^) that modulate immune checkpoints. These therapeutic interventions aim to inhibit STAT-mediated PD-L1 upregulation, thereby restoring immune surveillance and potentiating the efficacy of existing immunotherapies.

Therapeutic targeting of the PD-L1 and STAT pathway is a promising strategy because it directly disrupts a central mechanism of immune evasion in cancer. Inhibiting STAT signaling, particularly STAT3, which promotes PD-L1 expression on tumor cells, reactivates exhausted T cells and reduces immunosuppressive activity within the tumor microenvironment. This approach strengthens the immune system’s ability to recognize and eliminate cancer cells and may help overcome resistance to existing immunotherapies.^17^ In this section, we explore therapeutic strategies and methods designed to target these pathways, thereby enhancing anti-cancer efficacy (Figure 4, Table 1, and Table 2).

**Table 1:** Treatment options for targeting the STAT/PD-L1 axis in breast cancer

Name of Drug/Method	Type of treatment	Cell Line	Model of study	Effect on PD-L1	Effect on JAK/STAT	Effect on Cancer Cells	Reference
Everolimus	mTOR inhibitor	MCF-7	*In vitro* breast cancer cell line study	Significant decrease in PD-L1 (and CD155) expression	Marked inhibition of STAT3 phosphorylation (p-STAT3 reduced)	Dose-dependent inhibition of cell proliferation	Soltanshahi M. et al.^75^
Ibrutinib	BTK inhibitor	MCF-7	*In vitro* breast cancer cell line study	Significant decrease in PD-L1 (and CD155) expression	Significant inhibition of STAT3 phosphorylation	Dose-dependent inhibition of cell proliferation	
Stattic	Small-molecule STAT3 inhibitor	MCF-7 and T47D	*In vitro*	Decreases PD-L1 expression	Inhibits STAT3 phosphorylation at Y705; significantly reduces STAT1 activation and moderately reduces p65 activity	Increases CSC percentage and enhances EMT characteristics, suggesting that loss of STAT3’s inhibitory role on metastasis is unleashed	Ben-Yaakov H. et al.^51^
Low-dose SN-38	Small-molecule chemotherapeutic (active metabolite of irinotecan) with immunomodulatory properties	Mouse ID8 (ovarian cancer); human OVCA429; mouse 4T1-Luc (breast cancer)	*In vitro* cell culture; syngeneic mouse tumor xenograft models	Downregulates PD-L1 expression	Reduces phosphorylated STAT3 (pSTAT3) and associated cytokines (e.g., IL-6)	Inhibits tumor growth, increases apoptosis, enhances immune (NK and CD8^+^ T cell) infiltration, and improves overall survival	Chung YM. et al.^76^
Alisertib (Aurora A inhibitor)	Targeted chemotherapy/Immunomodulation	4T1 murine mammary carcinoma	*In vivo* studies using immune-competent murine models (e.g., WT BALB/c and MMTV-PyMT mice) and *in vitro* assays	Increases PD-L1 expression on MDSCs and tumor cells (which may potentially counteract T cell activity if unblocked)	Inhibits the JAK2/STAT3 pathway by reducing STAT3 phosphorylation, leading to decreased ROS production	Inhibits tumor growth by directly inducing apoptosis in immunosuppressive myeloid cells (MDSCs and TAMs) and indirectly enhancing T cell infiltration and activity against tumor cells	Yin T. et al.^77^
Alisertib + PD-L1 Blockade	Chemo-immunotherapy	4T1 murine mammary carcinoma	*In vivo* studies in murine models where alisertib was combined with PD-L1 monoclonal antibody treatment	PD-L1 blockade counteracts the alisertib-induced upregulation of PD-L1, thereby neutralizing its potential inhibitory effect on T cells	The inhibition of the JAK2/STAT3 pathway by alisertib is maintained, continuing to reduce immunosuppressive ROS production	The combination results in synergistic tumor inhibition, increased survival, and enhanced CD8^+^ T cell infiltration and functional activity, leading to improved overall antitumor efficacy compared	
GdNPs/Ce6-Ric	Combination SDT + HDAC6 Inhibition	4T1	*In vitro* & *in vivo* TNBC mouse model	Marked reduction in PD-L1 expression	Significant decrease in p-STAT3 (JAK/STAT inhibition)	Enhances apoptosis, significantly inhibits tumor growth and metastasis	Yu L. et al.^80^
Zr-CeO Nanozyme	ROS-scavenging nanozyme used as an immunomodulator	Renca, ACHN, 769P (renal cancer); 4T1 (TNBC)	Murine models of renal carcinoma and TNBC	Not directly targeted; effect not explicitly evaluated	Inhibits STAT3 activation (also affects ERK signaling)	No direct cytotoxicity; indirectly inhibits tumor growth by modulating immune cells	Mo W. et al.^81^
HA-PeiPLGA-MTX Nanoparticles (+PD-L1 Antibody)	PLGA-based nanocarriers loaded with methotrexate, coated with polyethyleneimine (Pei) and hyaluronic acid (HA), used in combination with a PD-L1 antibody	4T1 (murine breast cancer); RAW 264.7 (macrophages)	*In vitro* cell culture and *in vivo* orthotopic breast cancer model in BALB/c mice	Downregulation of PD-L1 expression in malignant cells	Downregulation of STAT3 (and NF-κB) signaling pathways	Induces apoptosis (upregulation of FADD, APAF-1, caspase-3; reduction of Bcl-2, MDR-1, survivin), reverses EMT (increased E-cadherin, decreased vimentin), reduces migration and metastasis	Cavalcante RS. et al.^82^
HA-TAT-TMC-TC NPs loaded with PD-L1/STAT3 siRNA	siRNA-mediated dual gene silencing (immunomodulatory therapy)	4T1 (breast cancer) and B16-F10 (melanoma); RAW 264.7 used for uptake and polarization studies	*In vitro* cell culture, *in vivo* tumor-bearing mouse models (BALB/C and C57BL/6J), and chorioallantoic membrane assay (CAM) assay	Approximately 80–90% reduction in PD-L1 mRNA and protein levels	Significant downregulation of STAT3 (affecting the JAK/STAT pathway) at mRNA and protein levels	Suppressed proliferation, migration, colony formation, and angiogenesis; enhanced apoptosis; reduced tumor size and weight; improved survival rate in mice	Bastaki S. et al.^83^
PPAB001	Bispecific antibody targeting CD47 and CD24	Human TNBC cell lines (MDA-MB-231, MDA-MB-468, BT549)	*In vitro* phagocytosis assays and 4T-1 syngeneic mouse model	No direct PD-L1 modulation; in combination, improves PD-L1 blockade efficacy	Activates JAK/STAT1 pathway when combined with Tecentriq	Enhances macrophage phagocytosis, delays tumor growth; moderate effect as a monotherapy	Yang Y. et al.^79^
Tecentriq (Atezolizumab)	PD-L1 immune checkpoint inhibitor	Primarily used in the *in vivo* model	4T-1 syngeneic mouse model	Directly blocks PD-L1; however, monotherapy may lead to increased M2 macrophage infiltration	No significant direct activation reported when used alone	Provides partial tumor growth inhibition; limited efficacy as monotherapy	
PPAB001 + Tecentriq	Combination immunotherapy (dual blockade of CD47/CD24 and PD-L1)	Human TNBC cell lines (*in vitro*) and 4T-1 syngeneic model (*in vivo*)	Both *in vitro* assays and *in vivo* mouse model	Significantly enhances PD-L1 blockade, overcoming immune-suppressive microenvironment	Strong activation of the JAK/STAT1 pathway leading to M1 polarization	Results in rapid and near-complete tumor regression, robust macrophage M1 polarization, and increased infiltration of cytotoxic CD8^+^ T cells in tumors	
M1-derived Exosomes + siSIRPα + siSTAT6	Combined siRNA delivery via exosomes targeting both SIRPα and STAT6	Same as above	*In vitro* macrophage reprogramming & 4T1 co-culture	When combined with anti-PD-L1, maximally removes inhibitory signals from both CD47-SIRPα and PD-L1 pathways	Combined downregulation shifts signaling toward STAT1/M1 profile	Synergistically enhances antitumor macrophage functions, achieving the highest phagocytic activity and strongest inhibition of 4T1 cell migration, invasion, and proliferation	Taghavi-Farahabadi M. et al.^85^
Anti-PD-L1 Antibody +M1-derived Exosomes + siSIRPα + siSTAT6	Immune checkpoint blockade therapy	4T1 breast cancer cells (in co-culture with treated macrophages)	*In vitro* co-culture (with various exosome/siRNA treatments)	Blocks PD-L1 on tumor cells, enhancing Fc receptor engagement and complementing exosome effects	Not directly altering JAK/STAT; supports overall reprogramming effect	Further boosts phagocytosis and antitumor effects, resulting in lower proliferation, migration, and invasion of tumor cells when combined with exosome-based treatments	
Dual CAR hPSC-NK Cells (Anti-PD-L1 + Anti-FITC CAR with FITC-folate adapter)	Cellular immunotherapy (adoptive NK cell therapy)	hPSC-derived NK cells (also tested in NK-92 cells)	*In vitro* co-culture with MDA-MB-231 tumor cells; *in vivo* xenograft mouse models	Engages tumor-expressed PD-L1, triggering receptor-mediated activation	Robust activation of STAT3/STAT5 signaling leading to enhanced proliferation and memory-like persistence	Increased tumor cell cytotoxicity, reduced tumor burden, and improved overall antitumor efficacy	Chang Y. et al.^86^
Self-reinforced Photodynamic Immunostimulator (PCS)	Combination nanotherapy integrating photodynamic therapy (PDT) with immunotherapy via PD-L1 blockade and STAT3 inhibition	Mouse breast cancer cells (4T1); Normal control: LO2; *In vivo*: 4T1 tumor-bearing mice and lung metastasis model	*In vitro* cell studies (4T1 uptake, ROS generation, ICD induction) and *in vivo* murine models (subcutaneous tumor and metastasis models)	Blocks and downregulates PD-L1 on tumor cells via a PD-L1 targeting peptide and enhanced uptake after PDT	Inhibits STAT3 phosphorylation (pSTAT3) through encapsulated Stattic, reducing pro-tumor signaling	Induces robust ROS generation under light (PDT), triggers immunogenic cell death (ICD), promotes apoptosis, and suppresses tumor growth and metastasis	Qiu Z. et al.^84^
Cardamonin	Natural flavonoid agent	MDA-MB-231 and MDA-MB-468 (TNBC cell lines)	*In vitro* (TNBC cell culture)	Decreased PD-L1 mRNA (74% reduction in MDA-MB-231; 84% reduction in MDA-MB-468) and protein (59% reduction in MDA-MB-231; complete inhibition in MDA-MB-468)	In MDA-MB-231: decreased JAK1 and STAT3; in MDA-MB-468: increased JAK1 and decreased STAT3	Induced dose-dependent cytotoxicity, morphological changes, and increased sensitivity	Mendonca P. et al.^87^
Delphinidin (Dp)	Natural anthocyanin agent	TNBC cell lines (MDA-MB-231 and BT-549); exosomes derived from TNBC cells; Jurkat cells in co-culture	*In vitro* (cell culture, co-culture, molecular docking, & dynamics simulations)	Decreased PD-L1 expression on TNBC cells and their exosomes, similar to the effect seen with Anti-PD-L1 treatment	Reduced phosphorylation of JAK2 and STAT3 (i.e., decreased p-JAK2/JAK2 and p-STAT3/STAT3 ratios)	Inhibited proliferation (dose-dependent; IC50 ∼103.6 μM for MDA-MB-231 and 93.27 μM for BT-549) and migration; restored T cell activity (increased CD69, IFN-γ, and TNF-β secretion)	Yu X. et al.^88^
Echinacoside (ECH)	Natural compound; used alone and with ICB agents	Breast and colorectal cancer models (tumor-bearing mice)	*In vitro* (cell studies) and *in vivo* (mouse model)	Downregulates IFN-γ-induced PD-L1 at both mRNA and protein levels via the JAK/STAT1/IRF1 signaling pathway	Inhibits the activation of the JAK/STAT1 pathway (reduced phosphorylation)	Reshapes tumor immune microenvironment by increasing effector T cell infiltration (IFN-γ^+^CD8^+^, Ki-67^+^CD8^+^), decreasing TIM-3^+^PD-1^+^ T cells, Tregs, and MDSCs; synergizes with anti-PD-1/CTLA-4 to enhance antitumor efficacy	Wang X. et al.^89^
Chitin	Natural polysaccharide used as a general CLP blocker	4T1-luc and 66cl4-luc TNBC intraductal mouse models; *in vitro* assays with RAW264.7 macrophages	*In vivo* (mouse intraductal TNBC models) and *in vitro* lymphatic adhesion/integration assays	Not directly modulated; used in combination with anti-PD-1 to overcome immunotherapy resistance	Reduced STAT3 phosphorylation (notably in high CLP-producing 4T1 tumors)	Reduced primary tumor growth and lymphatic metastasis; enhanced antitumor immunity and anti-PD-1 efficacy	Salembier R. et al.^90^
Isorhamnetin (ISO)	Natural flavonoid therapeutic agent	Canine mammary tumor U27 cells (WT and PD-L1 knockout) and murine 4T1 cells	*In vitro* assays, xenograft nude mouse model, and syngeneic BALB/c mouse model	Downregulates PD-L1 expression (demonstrated by immunofluorescence and western blot); reduced PD-L1 membrane presence	Inhibits phosphorylation of EGFR and STAT3; direct binding to EGFR confirmed by pull-down and SPR (KD ≈ 11.40 μM)	Inhibits cell growth, migration, and invasion; disrupts mitochondrial membrane potential; induces apoptosis (increased caspase-3, decreased Ki-67); suppresses tumor growth *in vivo*	Mei C. et al.^91^
Baicalein	Natural flavonoid agent	TNBC cell lines (e.g., 4T1, MDA-MB-231, BT549) co-cultured with adipocytes	*In vitro* adipocyte-tumor co-culture systems and murine xenograft/allograft models	Indirectly downregulates PD-L1 in tumor cells via reduced adipocyte-derived leptin	Inhibits p-STAT3 activation in TNBC cells	Enhances T cell-mediated tumor cell killing; suppresses tumor growth; improves overall antitumor immune response	Liu M. et al.^92^
β, β-Dimethylacrylshikonin	Natural naphthoquinone agent	TNBC cell lines (MDA-MB-231, Hs578T, BT-549)	*In vitro* assays and TNBC xenograft mouse models	Downregulates PD-L1 expression by inhibiting its transcription	Inhibits STAT3 Y705 phosphorylation and nuclear translocation; STAT3 overexpression reverses DMAS effects	Induces G2/M phase arrest, triggers mitochondria-dependent apoptosis, inhibits EMT-mediated migration/invasion, and potentiates paclitaxel efficacy while reducing immune evasion	Wu Z. et al.^93^
3,3′-Diindolylmethane (DIM)	Natural bioactive metabolite used alone and in combination with PD-1 blockade	*In vitro*: Bone marrow-derived MDSCs, splenic MDSCs, 4T1 cells; *in vivo*: 4T1 breast cancer and melanoma mouse models	*In vitro* assays (flow cytometry, qRT-PCR, western blot) and *in vivo* tumor-bearing mouse models	Reduces the immunosuppressive function and population of MDSCs, leading to increased CD4^+^/CD8^+^ T cell proliferation and elevated IFN-γ levels; enhances the efficacy of PD-1 blockade	Downregulates STAT3 phosphorylation via DIM-mediated reduction of miR-21 (with consequent upregulation of PTEN/PIAS3)	Inhibits tumor growth and metastasis by suppressing MDSC expansion and function; adoptive MDSC transfer reverses DIM effects	Sun Q. et al.^94^
Quercetin	Natural flavonoid; immunomodulator	MCF-10AT (precancerous), MCF-7 & MDA-MB-231 (breast cancer); γδ T cell co-culture	*In vitro* breast cancer and precancerous cell lines; immune cell co-culture assays	Decreased PD-L1 protein expression	Increased phosphorylation of JAK2 and STAT1	Induced apoptosis, inhibited proliferation, and enhanced synergistic killing by γδ T cells	Qiu D. et al.^95^
Apigenin	Natural flavonoid; immunomodulatory agent	Human: MDA-MB-468 (TNBC), SK-BR-3 (HER2+), HMEC; Mouse: 4T1 mammary carcinoma; MDA-MB-231 (constitutive PD-L1)	*In vitro* cell culture with IFN-γ/IFN-β stimulation; T cell co-culture assays	Inhibits IFN-γ/IFN-β-induced PD-L1 upregulation; no effect on constitutive PD-L1	Inhibits STAT1 phosphorylation at Tyr701 and Ser727	Enhances T cell-mediated responses (increased proliferation & IL-2 production); reduces immune evasion	Coombs MRP. et al.^54^

**Table 2:** Clinical trials of JAK/STAT-pathway-targeted agents combined with immune checkpoint inhibitors in breast cancer

NCT ID	Breast cancer subtype/setting	JAK/STAT-pathway-targeted agent	Immune checkpoint inhibitor	Phase/Design	Estimated/Actual Primary Completion	Current Status	Published Outcomes/Key Findings
NCT03012230	Metastatic TNBC. Heavily pretreated stage IV disease.	Ruxolitinib (JAK1/2 inhibitor)	Pembrolizumab (PD-1)	Phase I, dose-escalation	Estimated 2022–2023 (per registry history)	Completed	AACR 2024 abstract^96^: combination is *feasible*, with expected hematologic toxicities. Observed partial responses and instances of durable stable disease. Ongoing correlative analysis of JAK/STAT-immune signatures. Full manuscript not yet published.
NCT05967884	Early-stage ER+/HER2- invasive BC awaiting surgery (window-of-opportunity design).	Dupilumab (IL-4Rα blockade → inhibits IL-4/IL-13 → JAK1/3-STAT6 axis)	Cemiplimab (PD-1)	Phase II, randomized window of opportunity (WOO)	No official completion date posted; small 2-arm WOO trial (initiated 2023)	Not yet recruiting/ongoing	Early SITC/JITC 2024 pre-op data^97^: combination reduces Th2-STAT6 signaling and enhances IFN/antigen-presentation signatures vs. PD-1 alone. Short-course safety acceptable. Pathologic response data not yet available.
NCT02646748*(Basket trial including breast cancer)*	Advanced/metastatic solid tumors, including a breast cancer cohort (non-separately reported). Provides breast-relevant safety/pharmacodynamic data.	Itacitinib (selective JAK1 inhibitor) and/or Parsaclisib (PI3Kδ inhibitor)	Pembrolizumab (PD-1)	Phase I, multi-arm dose escalation & expansion	Completed (results published 2023)	Completed; combination development in unselected solid tumors discontinued	Phase I data^98^: combinations showed acceptable safety across tumor types, including breast cancer cases. Modest antitumor activity overall; no compelling signals to continue broad development. Offers foundational evidence on safety, pharmacokinetics (PK), and immune-modulation when combining JAK1 inhibition with PD-1 blockade in solid tumors, including breast cancer.

### Clinical trials

The clinical evidence evaluating JAK/STAT-pathway-targeted therapies in combination with immune checkpoint inhibitors in breast cancer remains highly limited. The phase I trial of ruxolitinib plus pembrolizumab in metastatic TNBC (NCT03012230) demonstrated that the combination is feasible, with manageable hematologic toxicity and early signals of clinical activity, including partial responses and durable stable disease in a subset of heavily pretreated patients; however, detailed correlative analyses and mature efficacy data have not yet been published.^96^ The ongoing window-of-opportunity trial evaluating cemiplimab with or without dupilumab (IL-4Rα blockade targeting the JAK-STAT6 axis) in early ER^+^/HER2- breast cancer (NCT05967884) has thus far reported only preliminary immunologic findings, showing enhanced interferon-related and antigen-presentation signatures in the combination arm, without available clinical response endpoints.^97^ A broader phase I basket study combining itacitinib (JAK1 inhibitor) or parsaclisib with pembrolizumab (NCT02646748) included a breast cancer cohort but did not report breast-specific outcomes; overall, the combinations demonstrated acceptable safety but modest antitumor activity across tumor types. Collectively, these studies highlight that clinical investigation of JAK/STAT-targeted combinations with immunotherapy in breast cancer is still at a very early stage, with limited sample sizes, incomplete reporting, and minimal efficacy data, underscoring a significant translational gap and the need for more robust, breast-cancer-focused clinical trials (Table 2).^98^

### Synthetic drugs

#### Everolimus combined with ibrutinib

Everolimus, an mTOR inhibitor, and ibrutinib, a BTK inhibitor, are small-molecule agents that disrupt key signaling pathways in cancer cells. In MCF-7 breast cancer cells, treatment with everolimus and ibrutinib, administered individually or in combination, produced significant antiproliferative effects. These treatments reduced mRNA expression of the immune checkpoint ligands PD-L1 and CD155, whereas their combined use increased galectin-9 expression. Both drugs also decreased STAT3 phosphorylation, which showed a positive correlation with PD-L1 and CD155 expression and a negative correlation with galectin-9 levels. These results indicate that targeting the mTOR and BTK pathways with everolimus and ibrutinib can modulate immune evasion mechanisms in breast cancer and may enhance antitumor immune responses.^75^

#### AZD1775

AZD1775, also known as adavosertib, is a selective small-molecule inhibitor of WEE1, a tyrosine kinase that plays an essential role in cell cycle regulation. WEE1 primarily phosphorylates and inhibits cyclin-dependent kinase 1 (CDK1), preventing cells that have sustained DNA damage from entering mitosis prematurely.^99^ Combining the WEE1 inhibitor AZD1775 with a single high dose of irradiation significantly delays tumor growth in breast cancer models by strengthening antitumor immunity. The antitumor effect depends on CD8^+^ T cells, and AZD1775 enhances T cell-mediated cytotoxicity *in vitro* and *in vivo*. The combination increases CD8^+^ T cell infiltration, elevates granzyme B expression, and promotes dendritic cell activation, which supports improved T cell priming. It also reshapes the tumor microenvironment by reducing MDSCs and regulatory T cells. In addition, the treatment lowers PD-L1 expression on tumor cells through inhibition of the STAT3 to IRF1 to PD-L1 signaling pathway.^100^

#### Stattic

Researchers investigated how STAT3 and p65 regulate cancer stem cell dynamics in hormone receptor-positive and HER2-negative breast cancer. In a simulated tumor microenvironment containing estrogen, TNFα, and EGF, cells showed increased abundance of cancer stem cells, EMT features, and higher levels of CXCL8 and PD-L1. Pharmacologic inhibition of STAT3 with Stattic blocked Y705 phosphorylation but unexpectedly increased cancer stem cell enrichment and EMT traits, even as CXCL8 and PD-L1 expression decreased. Genetic deletion of STAT3 did not reproduce these effects, indicating compensation by other pathways. Subsequent experiments implicated NF-κB p65 as a compensatory driver, as its inhibition also increased cancer stem cell abundance. Dual inhibition of STAT3 and p65 further intensified cancer stem cell enrichment and chemoresistance. Clinical data from luminal A breast cancer patients showed that higher phosphorylation of STAT3 and p65 correlated with lower cancer stem cell gene signatures and more favorable outcomes, suggesting that balanced activity of these transcription factors helps restrain cancer stem cell-driven tumor aggressiveness.^51^

#### SN-38

SN-38, the active metabolite of irinotecan, modulates the tumor immune microenvironment at low doses by suppressing STAT3 signaling. Because STAT3 activation typically increases PD-L1 expression and promotes immune escape, inhibition of STAT3 by SN-38 leads to a significant reduction in PD-L1 on tumor cells. This reduction improves tumor recognition by the immune system and enhances the effectiveness of anti-PD-1 therapy. By limiting PD-L1-mediated suppression, SN-38 increases the activation of cytotoxic CD8^+^ T cells and NK cells, resulting in a stronger antitumor immune response.^76^

#### Alisertib

Alisertib, a selective Aurora A kinase inhibitor, acts on both tumor cells and the immune microenvironment to enhance antitumor responses. In addition to blocking cancer cell proliferation, it induces apoptosis in immunosuppressive myeloid populations such as MDSCs and TAMs. Alisertib disrupts STAT3 dependent signaling in these cells, which lowers reactive oxygen species (ROS) production and reduces their capacity to suppress T cell activity. This shift promotes greater infiltration and activation of antitumor T lymphocytes. When combined with anti PD-L1 therapy, alisertib strengthens the overall immune response by converting the tumor microenvironment from immunosuppressive to immunostimulatory, resulting in improved tumor control.^77^

#### MEKi

Loss/heterogeneous downregulation of MHC-I in breast cancer (most commonly in triple-negative breast tumors) dysregulates CD8^+^ T cell recognition/cytotoxicity and is a major mechanism of resistance to anti-PD-L1 therapy. In general, blockade of PD-L1 reinvigorates exhausted T cells by restoring effector function directed against tumor antigens, which requires sufficient MHC-I levels to allow for antigen presentation. Deficiency of MHC-I thus leads to immune “cold” phenotypes with T cell exclusion and suboptimal therapeutic response. As such, MHC-I deficiency in breast cancer is emerging as a critical checkpoint in immunotherapy resistance.^101^ MEK inhibition alters the MAPK signaling cascade, which generally limits STAT activation, leading to increased phosphorylation and activity of STAT1, STAT3, and STAT5. Enhanced STAT signaling promotes the transcription of genes that elevate MHC-I expression on breast cancer cells, thereby improving antigen presentation and increasing tumor visibility to the immune system. MEK inhibitors also induce PD-L1 upregulation, which can contribute to immune evasion by suppressing T cell activity. However, this rise in PD-L1 becomes therapeutically advantageous when combined with immune checkpoint inhibition, as blocking PD-L1 under these conditions enables a more potent antitumor immune response. Together, these effects increase the immunogenicity of breast tumors by improving antigen presentation and creating opportunities for more effective immunotherapy strategies that rely on coordinated modulation of MHC-I and PD-L1.^78^

#### PPAB001

PPAB001 is a novel bispecific antibody fusion protein that simultaneously targets CD47 and CD24, two key “don’t eat me” signals frequently overexpressed in TNBC cells. By blocking these signals, PPAB001 enhances macrophage phagocytosis of tumor cells, effectively overcoming one of TNBC’s critical immune evasion strategies. Moreover, when combined with anti-PD-L1 therapy, PPAB001 reprograms TAMs toward a pro-inflammatory M1 phenotype. This reprogramming is mediated by the upregulation of the CXCL9/10-CXCR3 axis and the subsequent activation of the JAK/STAT1 signaling pathway, thereby amplifying the antitumor immune response and improving the overall efficacy of PD-L1 blockade in treating TNBC.^79^

### Nanoparticles

Nanomaterials are crucial in tumor immunotherapy because they serve as smart carriers that enhance the delivery and efficacy of immune drugs. They can protect and concentrate antigens, mRNA, and immune modulators in the tumor microenvironment, enhance T cell and NK cell responses, overcome immunosuppression, and reduce side effects, making immunotherapy more potent and precise, even though clinical translation is still challenging.^102^

#### GdNPs/Ce6-Ric

GdNPs/Ce6-Ric nanoparticles combine the sonosensitizer Chlorin e6 with the HDAC6 inhibitor Ricolinostat within pH-responsive gadolinium-based mesoporous carriers that preferentially accumulate in tumors and release their payload in the acidic tumor microenvironment. Ultrasound activation of Chlorin e6 produces ROS that trigger immunogenic cell death and promote dendritic cell maturation and cytotoxic T cell activation. At the same time, Ricolinostat inhibits the HDAC6/pSTAT3/PD-L1 pathway, reducing PD-L1 expression, enhancing the infiltration and activity of NK cells and cytotoxic T cells, and decreasing regulatory T cells. Through this combined mechanism, the nanoparticles generate a strong antitumor immune response and suppress tumor growth and metastasis in TNBC.^80^

#### ROS-scavenging nanozymes

Zr-CeO ROS-scavenging nanozymes act as synthetic antioxidant enzymes that reduce excessive ROS in the tumor microenvironment. In breast cancer, lowering ROS levels weakens immunosuppression by reducing the unfolded protein response in MDSCs and decreasing the expression of arginase 1 and iNOS, which normally suppress T cell proliferation. The nanozymes also block ROS-dependent ERK and STAT3 signaling in TAMs and prevent their shift toward the pro-tumorigenic M2 phenotype. Through these mechanisms, the nanozymes disrupt immunosuppressive networks and improve sensitivity to immune checkpoint blockade, including anti-PD-1 therapy, ultimately enhancing antitumor immunity.^81^

#### poly(lactic-co-glycolic acid) nanocarriers

HA PeiPLGA MTX nanoparticles are designed to target breast cancer cells that overexpress HA receptors and to deliver methotrexate directly to the tumor. When combined with a PD-L1 antibody, they not only improve chemotherapeutic delivery but also modulate the tumor immune microenvironment. The nanoparticles inhibit STAT3 and NF-κB signaling, shift TAMs from the immunosuppressive M2 state to the pro-inflammatory M1 state, reduce PD-L1 expression on tumor cells, and promote apoptosis while limiting migration and metastasis. Through these combined effects, the system strengthens antitumor immunity and improves therapeutic outcomes in breast cancer models.^82^

#### Hyaluronate-TAT-TMC-TC

Hyaluronate-TAT-TMC-TC nanoparticles are a multifunctional gene delivery system composed of N,N,N-trimethyl chitosan (TMC) and thiolated chitosan (TC) conjugated with the cell-penetrating TAT peptide and coated with hyaluronic acid (HA). This design enhances siRNA encapsulation, stability, and targeted delivery to CD44-overexpressing cancer cells. These nanoparticles were loaded with siRNAs against PD-L1 and STAT3, leading to an 80–90% reduction in both mRNA and protein levels of these key immunosuppressive and oncogenic molecules. Consequently, dual silencing of PD-L1 and STAT3 significantly reduced tumor size and weight in tumor-bearing mice by suppressing cancer cell proliferation, migration, and angiogenesis, while also inducing robust antitumor immune responses. Cellular uptake was confirmed via confocal microscopy and flow cytometry using CY5-labeled siRNA, which demonstrated efficient internalization mediated by the HA and TAT components. These findings support the potential of this nanoparticle-based strategy as a promising immunomodulatory therapy in cancer treatment.^83^

#### Photodynamic immunostimulator

The self-reinforced photodynamic immunostimulator (PCS) is a nanosystem that integrates tumor-targeted photodynamic therapy and STAT3 inhibition to simultaneously treat metastatic breast cancer. The PCS comprises a PD-L1-targeting chimeric peptide and the STAT3 inhibitor Stattic. The peptide directs PCS to PD-L1-overexpressing tumor cells, where light-induced PpIX photosensitizer production of ROS results in immunogenic cell death. The STAT3 inhibitor Stattic inhibits STAT3 phosphorylation and reduces intracellular PD-L1 expression. The combination of these two mechanisms results in the blockage of PD-L1 and PD-1 signaling, the restoration of CD8^+^ T cell function, and a tumor microenvironment amenable to the induction of robust antitumor immunity, which, in turn, suppresses primary tumor growth and metastasis.^84^

### Exosomes

The M1-derived exosomes, engineered to deliver siRNAs targeting SIRPα and STAT6 to M2 macrophages, successfully reprogrammed M2 macrophages into M1-like macrophages. The exosomes from M1 macrophages slightly enhanced the antitumor function, and the treatment with exosomes pre-loaded with siSTAT6 and/or siSIRPα further reduced the expression of key M2 pro-tumor signals. *In vitro*, macrophages treated with exosomes with these siRNAs showed a significant increase in phagocytic function, which was further augmented when combined with an anti-PD-L1 antibody. The anti-PD-L1 antibody blocked the inhibitory signaling from tumor cells and increased Fc receptor-mediated activation. The combination of these therapies led to macrophages exerting a potent antitumor effect by reducing the migration, invasion, and proliferation of 4T1 breast cancer cells.^85^

### CAR-NK Cells

Engineering chimeric antigen receptors (CARs) with dual targeting functionality, integrating an anti-PD-L1 nanobody alongside a universal anti-FITC single-chain variable fragment, offers a promising strategy to enhance both the effectiveness and safety of NK cell-based immunotherapy. These next-generation CAR constructs incorporate NK cell-specific signaling domains, including the NKG2D transmembrane region and the 2B4 co-stimulatory domain, as well as a truncated IL-2 receptor β-chain fused with a STAT3-binding YXXQ motif. This design promotes robust, antigen-dependent activation of STAT3 and STAT5 signaling, leading to increased NK cell proliferation, improved functional persistence, and the acquisition of memory-like properties. These enhancements result in stronger immunological synapse formation with tumor cells and significantly greater cytotoxicity compared with unmodified NK cells. *In vivo*, dual-targeting chimeric antigen receptor-natural killer cells (CAR-NK) cells demonstrated prolonged survival and sustained antitumor responses. When derived from human pluripotent stem cells (hPSCs), this platform enables standardized, scalable production and precise genetic engineering, positioning these CAR-NK cells as a potent off-the-shelf immunotherapy for targeted cancer treatment.^86^

### Natural products

Natural products are an essential source for identifying potential modulators of breast cancer immunotherapy. Targeting multiple tumor-promoting and/or immune-regulatory pathways, natural products exhibit broad-spectrum bioactivities, including antitumor and antimetastatic effects. Many of these natural agents have also been shown to reprogram tumor-infiltrating immune cells by both activating CD8^+^ T cells, NK cells, and dendritic cells and inhibiting regulatory populations, such as Tregs and MDSCs. Furthermore, natural products have been reported to downregulate the expression of immune checkpoints, such as PD-L1, and to reinvigorate antitumor immunity. The broad diversity of natural product structures also enables them to bind and modulate a variety of molecular targets, including many crucial signaling pathways known to promote breast cancer progression. These features, in combination with their relative safety in comparison with cytotoxic chemotherapeutics, make natural products an attractive option for drug combinations to potentiate.^103^^,^^104^

#### Cardamonin

Cardamonin, a chalcone flavonoid, is a well-known phytochemical with potent anticancer activity. It has been shown to have robust immunomodulatory activity in TNBC. Cardamonin significantly decreased PD-L1 expression at both the mRNA and protein levels in MDA-MB-231 and MDA-MB-468 cells. Cardamonin also inhibited the JAK/STAT pathway, as shown by decreased JAK1 and STAT3 expression in MDA MB 231 cells and a selective reduction in STAT3 in MDA MB 468 cells (JAK1 was increased in this cell line). The decreased viability and altered inflammatory cytokine profile associated with morphologic features of enhanced immune activation can be traced back to these molecular alterations. Taken together, these data suggest that cardamonin may sensitize TNBC to immunotherapy through PD-L1 suppression and JAK/STAT modulation.^87^

#### Delphinidin

Delphinidin (Dp), a bioactive anthocyanin abundant in deeply colored fruits and vegetables, has been shown to have potent anticancer activity. Dp was found to repress PD-L1 expression on the surface of TNBC cells and on tumor-derived exosomes, thereby suppressing tumor growth. The underlying mechanism was thought to be related to the inhibition of JAK2/STAT3 signaling pathway, which was supported by the reduced phosphorylation of JAK2 and STAT3. Moreover, delphinidin not only inhibited TNBC cell proliferation and migration, but also restored the function of T cells in a co-culture system, as demonstrated by upregulation of CD69 and secretion of IFN-γ and TNF-β by Jurkat T cells. Taken together, these results established the potential of delphinidin in remodeling the tumor immune microenvironment and served as a rationale for its use as a combinatorial agent to augment the efficacy of immunotherapies in TNBC.^88^

#### Echinacoside

Echinacoside is an endogenous phenylethanoid glycoside that inhibits tumor immune escape in breast and colorectal cancer by inhibiting inducible PD-L1 expression via the JAK to STAT1 to IRF1 signaling pathway. Echinacoside inhibited PD-L1 transcription and protein levels after stimulation with IFN-γ and promoted T cell activation. Treatment in tumor-bearing mice led to increased infiltration of functional CD8^+^ T cells, including IFN-γ^+^ and Ki-67^+^ subsets, and decreased immunosuppressive populations, including exhausted T cells, regulatory T cells, and MDSCs. Echinacoside combined with anti-PD-1 or anti-CTLA-4 antibodies produced synergistic antitumor effects without additional toxicity, suggesting that it may be an effective means to improve response to immunotherapy.^89^

#### Chitin

Chitin, a natural polysaccharide and general blocker of chitinase-like proteins such as CHI3L1 and CHI3L3, counteracts immunosuppression in TNBC. In intraductal mouse models, chitin reduced the production of immunosuppressive proteins and lowered STAT3 phosphorylation, leading to a more active tumor immune microenvironment. Treatment decreased primary tumor growth and lymphatic metastasis and enhanced the efficacy of anti-PD-1 therapy by increasing infiltration and activation of antitumor T cells. These findings support chitin as a potential immunomodulatory agent for enhancing checkpoint blockade in TNBC.^90^

#### Isorhamnetin

Isorhamnetin is a natural flavonoid that inhibits the EGFR to STAT3 to PD-L1 pathway and shows anticancer activity in canine mammary tumors. In U27 cells, isorhamnetin reduced PD-L1 expression as confirmed by immunofluorescence and western blot analysis. CRISPR-mediated PD-L1 knockout demonstrated that the cytotoxic effects of isorhamnetin are partly dependent on PD-L1 downregulation. The compound also reduced migration and invasion, disrupted mitochondrial membrane potential, and induced apoptosis. *In vivo*, isorhamnetin suppressed tumor growth in both U27 xenograft models and 4T1 syngeneic models and was associated with lower levels of phosphorylated EGFR and STAT3. Pull-down and surface plasmon resonance assays confirmed that isorhamnetin directly binds to EGFR, identifying it as a key target within this signaling axis.^91^

#### Baicalein

Baicalein is a natural flavonoid with multiple antitumor effects and enhances antitumor immunity in TNBC by targeting the tumor microenvironment. Baicalein suppresses leptin production in adipocytes by downregulating the SREBP1 pathway. Because adipocyte-derived leptin normally increases PD-L1 expression in TNBC cells through activation of phosphorylated STAT3, reducing leptin leads to lower PD-L1 and pSTAT3 levels in tumor cells. This decreases immune escape, strengthens T cell-mediated tumor killing, and suppresses tumor growth, with powerful effects in obesity-associated breast cancer.^92^

#### β, β-Dimethylacrylshikonin

β,β-Dimethylacrylshikonin (DMAS) is a naphthoquinone compound with strong antitumor activity in TNBC. DMAS suppresses cell proliferation by inducing G2 and M phase arrest and promoting intrinsic mitochondrial apoptosis, while also reducing migration and invasion by reversing EMT. At the molecular level, DMAS selectively inhibits STAT3 phosphorylation at Tyr705, blocking its nuclear translocation and preventing PD-L1 transcriptional activation. STAT3 overexpression weakens the antitumor effects of DMAS, whereas STAT3 knockdown reproduces them, demonstrating that DMAS acts through a STAT3-dependent mechanism. *In vivo*, DMAS reduces tumor growth and metastasis, increases the therapeutic benefit of paclitaxel, and lowers PD-L1 expression.^93^

#### 3,3′-Diindolylmethane

3,3′-Diindolylmethane (DIM), a metabolite derived from cruciferous vegetables, enhances the antitumor activity of PD-1 blockade by targeting MDSCs. DIM reduces the expansion of these cells and diminishes their immunosuppressive function by lowering the expression of arginase 1 and iNOS and partially restoring T cell proliferation. Mechanistically, DIM decreases miR-21 levels in MDSCs, which increases the expression of PTEN and PIAS3, two negative regulators of STAT3, and reduces STAT3 phosphorylation. *In vivo*, DIM inhibited 4T1 tumor growth by reducing MDSC populations throughout multiple tissues and improved the response to PD-1 antibody therapy, as shown by greater CD4^+^ and CD8^+^ T cell abundance and higher intratumoral IFN-γ levels. Adoptive transfer and genetic studies confirmed that the antitumor effects of DIM depend on modulation of the miR-21 to STAT3 pathway in MDSCs.^94^

#### Quercetin

Quercetin has both direct anticancer effects and immunomodulatory activity in breast cancer. It inhibits proliferation and induces apoptosis in precancerous and malignant breast cells in a time- and concentration-dependent manner. At the same time, quercetin promotes the differentiation of human γδ T cells toward the Vδ2 subset, which increases their cytotoxicity against breast cancer cells. Mechanistically, quercetin activates the JAK and STAT1 pathway by increasing IFN-γ receptor expression and phosphorylation of JAK2 and STAT1, while reducing PD-L1 levels. These combined actions enhance immune-mediated tumor cell killing and support the potential of quercetin as both a preventive agent for precancerous breast lesions and an adjuvant therapy for breast cancer.^95^

#### *Taraxacum mongolicum* extract

*T. mongolicum* extract, also known as dandelion extract, is a natural compound obtained from a traditional Chinese medicine plant that has been used for centuries to treat breast-related disorders. In a TNBC cell line microenvironment with TAMs, the extract showed potent immunomodulatory and antitumor effects. It inhibited the immunosuppressive IL-10/STAT3/PD-L1 axis by blocking STAT3 activation and downregulating PD-L1 expression on tumor cells. This suppression of PD-L1 limited the malignant properties of TNBC cells, including proliferation, migration, and invasion. Furthermore, the dandelion extract induced the repolarization of TAMs from the M2 immunosuppressive phenotype to the M1 pro-inflammatory phenotype, thereby enhancing antitumor immune responses. These findings highlight its potential as a novel immunotherapeutic approach for TNBC.^105^

#### Apigenin

Apigenin is a dietary flavonoid that is abundant in fruits, vegetables, and traditional medicinal herbs. Apigenin is known for its anticancer and immunoregulatory effects. Apigenin inhibited IFN-γ-induced PD-L1 expression in several breast cancer cell lines, including triple-negative MDA-MB-468, HER2-positive SK-BR-3, and murine 4T1 cells. Mechanistically, apigenin interfered with STAT1 signaling by preventing the phosphorylation of key activation residues (Tyr701 and Ser727). This action led to the abrogation of STAT1-induced PD-L1 transcriptional upregulation. The reduction in PD-L1 led to decreased association with PD-1 on T cells and reduced T cell inhibition. In co-culture with T cells, this effect led to increased T cell proliferation and higher IL-2 production. These data support a role for apigenin in regulating oncogenic signaling in tumor cells and augmenting antitumor immune responses.^54^

#### Sepiapterin

Sepiapterin, a precursor of tetrahydrobiopterin, alters arginine metabolism in breast tumors by restoring BH4 levels and shifting metabolism from polyamine synthesis toward nitric oxide production. The resulting increase in nitric oxide suppresses tumor cell proliferation and lowers PD-L1 expression by inhibiting STAT3 signaling. Sepiapterin also reshapes the immune microenvironment by converting TAMs from an M2-like immunosuppressive phenotype to an M1-like proinflammatory state. This shift enhances T cell-mediated antitumor responses and may improve the effectiveness of immune checkpoint therapies.^106^

## Conclusion

Conclusively, this review has underlined that the JAK/STAT pathway, predominantly STAT1 and STAT3, plays a pivotal role in PD-L1 expression and the immune contexture of breast cancer. A multitude of stimuli have been found to induce PD-L1 expression through this axis, such as inflammatory cytokines, hypoxia, metabolic reprogramming, and exosomal signals, in both the tumor cells and the immune and stromal components of the tumor microenvironment, with chronic activation of STAT signaling promoting immune evasion, tumor progression, and resistance to immune checkpoint blockade therapy, particularly in more aggressive subtypes such as TNBC. Additionally, the recent literature has provided numerous therapeutic insights to target this axis. JAK or STAT inhibitors, natural compounds, RNA-based therapeutics, and nanomedicines have shown potential to downregulate PD-L1 expression, reprogram the tumor immune microenvironment, and augment antitumor immunity. Combination strategies targeting STAT1 and STAT3, or combining STAT1/STAT3 inhibition with chemotherapy, targeted agents such as Herceptin, or photodynamic therapy, have shown promising efficacy in preclinical models by not only inhibiting tumor-intrinsic immune evasion mechanisms but also reinvigorating cytotoxic lymphocytes. This has opened new avenues for more effective and durable immunotherapeutic responses, with the JAK/STAT-PD-L1 axis being an attractive target given its role in both tumor and immune cells. However, the intricacy of this pathway and its context-dependent behavior also poses a challenge. For instance, while STAT3 inhibition could reverse immunosuppression, non-selective targeting could also impair crucial immune cell functions. Additionally, redundancy or compensatory mechanisms in other signaling pathways, along with the inherent intratumoral and intertumoral heterogeneity, could limit the efficacy of single-agent therapies. Crosstalk between the JAK/STAT pathway and PD-L1 has been documented in multiple tumor types. However, breast cancer exhibits several unique regulatory features. First, hormone-receptor signaling (especially ER) can suppress PD-L1 expression, making PD-L1 regulation highly subtype dependent and most pronounced in TNBC. Second, BRCA1/2 deficiency and PARP-inhibitor-induced DNA damage activate the cGAS/STING and type I/II IFN/JAK/STAT pathways, establishing a distinct link between homologous recombination defects and PD-L1 upregulation. Third, oncogenic drivers differ by subtypes such as HER2-PI3K/AKT/mTOR signaling or basal-like EGFR/STAT3 activity, resulting in subtype-specific cooperation with JAK/STAT in modulating PD-L1. Finally, TNBC demonstrates marked MHC-I heterogeneity and unique NK/T cell spatial patterns, which influence PD-L1 induction in both tumor and immune cells. Together, these factors make PD-L1 regulation in breast cancer more heterogeneous and context-dependent than in many other solid tumors.

In breast cancer, several PD-L1 regulatory mechanisms are supported by strong and consistent evidence. The most robust findings involve IFN-γ/JAK/STAT1/3 signaling, where pathway activation, JAK2/PD-L1 locus amplification, and cytokine stimulation directly enhance PD-L1 transcription, and JAK/STAT inhibition reliably suppresses it. Another well-validated category is oncogenic RTK signaling, including EGFR-, HER2-, and FGFR2-driven pathways, which upregulate PD-L1 through downstream STAT3, AKT/mTOR, or ERK activation. In addition, epigenetic and ncRNA-mediated mechanisms, such as HDAC2-facilitated STAT1 promoter binding and the STAT1/TINCR/miR-199a-5p/USP20 axis affecting PD-L1 stability, have been repeatedly demonstrated in breast cancer models. Microenvironmental factors like hypoxia, metabolic stress, and inflammatory cytokines also elevate PD-L1 through HIF-1α, AKT/STAT3, and TAM-associated STAT3 signaling. Finally, post-translational regulation, especially PD-L1 glycosylation that stabilizes the protein and reinforces STAT1/3 signaling, along with PD-L1-containing extracellular vesicles, represents another well-established mechanism. Collectively, the mechanisms with the strongest evidence in breast cancer are JAK/STAT-mediated transcriptional activation, RTK-oncogenic signaling, epigenetic/ncRNA modulation, microenvironmental STAT3-based induction, and glycosylation-dependent post-translational stabilization.

Breast cancer subtypes, including luminal A, luminal B, HER2-positive, and triple-negative, exhibit profoundly different immune contextures that shape PD-L1 biology and immunotherapy responsiveness. Luminal tumors typically display lower baseline immune infiltration, with PD-L1 expression largely confined to tumor-associated immune cells, whereas HER2-positive cancers show cytokine-driven PD-L1 induction, partly mediated by HER2-linked STAT3 activation. In contrast, TNBC is the most immunogenic subtype, characterized by abundant CD8^+^ T cell infiltration, frequent IFN-γ-induced JAK/STAT activation, and high PD-L1 expression across cancer cells, macrophages, dendritic cells, and even stromal components. This review highlights multiple cellular sources of PD-L1, including tumor cells, TAMs, CD8^+^ T cells, endothelial cells, and exosome-carrying compartments. It details molecular mechanisms such as IFN-γ/JAK/STAT signaling, EGFR/STAT3 hyperactivation, metabolic stress, hypoxia-HIF1α signaling, extracellular vesicle-mediated STAT3 induction, and chromatin remodeling that drive PD-L1 upregulation. These subtype-specific mechanisms influence therapeutic response: PD-L1 is a robust predictive biomarker in TNBC, moderately informative in HER2-positive disease, and less reliable in luminal cancers where immunotherapy benefit is limited. Overall, understanding how each subtype differentially regulates PD-L1 provides a framework for optimizing immune checkpoint blockade strategies across heterogeneous breast cancer populations.

Additionally, integrating JAK/STAT inhibition with immune checkpoint blockade, chemotherapy, or other immunomodulatory agents in a context-specific manner could enhance efficacy and overcome resistance. The importance of JAK/STAT signaling extends well beyond its role in modulating PD-1/PD-L1 checkpoint blockade and should be emphasized in the context of emerging immunotherapy combinations, particularly those involving DNA damage, targeted agents such as PARP inhibitors. PARP inhibition activates cGAS/STING and type I interferon pathways in BRCA1/2-deficient tumors, leading to robust JAK/STAT-driven transcription of interferon-stimulated genes and PD-L1. This creates a therapeutically exploitable state in which JAK/STAT activation enhances tumor immunogenicity while simultaneously generating adaptive resistance through PD-L1 upregulation. Importantly, BRCA1/2-mutant tumors display distinct JAK/STAT profiles and interferon responsiveness, which influence their differential sensitivity to PARP inhibitor-immunotherapy combinations. Thus, integrating JAK/STAT-targeted approaches with PARP inhibitors not only amplify antitumor immunity but also prevent compensatory immunosuppressive signaling, highlighting the pathway’s central role in designing next-generation combination immunotherapies.

### Limitations of the study

Future studies will need to use the rich mechanistic data on JAK/STAT dysregulation described here to inform rational approaches to biomarker development and usage in clinical trials that allow patient selection and dynamic adaptation of immunotherapy treatment in breast cancer. Distinct genomic (copy-number alterations of JAK2 or upstream receptors), transcriptional (STAT1/3/4 pathway scores), post-translational (phospho-STAT1/3, PD-L1 glycosylation), and microenvironmental (IL-6/IL-10-rich, STAT3-driven) features of JAK/STAT dysregulation can be interrogated at the single cell level or as part of composite signatures for biomarker discovery rather than single readouts such as PD-L1 immunohistochemistry (IHC) alone. Integrating these diverse features into more informative and dynamic predictors may allow identification of breast cancer patients who will most likely respond to PD-1/PD-L1 blockade alone, JAK/STAT inhibitors, or rational combinations (e.g., with PARP inhibition, radiotherapy, or other targeted agents) and guide appropriate sequencing or combination strategies (as reviewed in ref. 101). Longitudinal measurement of JAK/STAT pathway activation, relative to baseline and at multiple time points after treatment initiation in on-treatment biopsies, may also capture the mechanisms of adaptive resistance (e.g., IFN-γ-driven STAT1/STAT3 upregulation of PD-L1 or IL-6/STAT3-mediated CD8^+^ T cell dysfunction) and support early decisions to escalate or switch treatment.

Beyond tumor cell-intrinsic changes, JAK/STAT-associated biomarkers in the tumor microenvironment and peripheral blood are likely to be essential for guiding treatment decisions. The presence of STAT3-high, M2-polarized macrophages, IL-6/IL-10-driven T cell reprogramming, γδ Treg cells that induce STAT3 activation in dendritic cells, and obesity-associated STAT3-dependent metabolic exhaustion of CD8^+^ T cells in breast tumors all point to the relevance of “JAK/STAT immune states” in the tumor microenvironment. These may be quantified using multiplex IHC, spatial transcriptomics, or single-cell RNA-sequencing and included in the clinical decision-making algorithms for breast cancer immunotherapy. Circulating biomarkers, such as serum IL-6/IL-10, sEV cargo (JAK/STAT-regulated miRNAs or PD-L1^+^ vesicles), and phosphorylated STATs in peripheral immune cell subsets, could serve as minimally invasive pharmacodynamic biomarkers to monitor pathway targeting and early detection of emerging resistance mechanisms. At the same time, the JAK/STAT-related genomic contexts (e.g., BRCA1/2 and broader DDR defects, FGFR2 activation, or IL20RA overexpression) described above need to be prospectively evaluated in clinical trials as stratification variables for JAK/STAT inhibitors, combination agents, or immunotherapies, to determine whether specific mutational signatures represent actionable contexts for JAK/STAT-dependent and immunotherapy-responsive phenotypes.

To enable translation of these ideas into the clinic, future clinical trials must consider the incorporation of comprehensive multi-omic JAK/STAT profiling, including genomics, phospho-proteomics, transcriptomic pathway scores, and circulating tumor DNA, microRNA, and EV readouts, along with the standardized assessment of PD-L1, tumor-infiltrating lymphocytes (TIL) quantification, and other biomarkers (such as TMB) that will be essential to measure the contribution of each feature and test their incremental value on top of existing algorithms. Adaptive trial designs that prospectively test novel JAK/STAT-targeting agents (including small-molecule inhibitors, RNA-based therapeutics, or nanomedicine drug delivery systems) in biomarker-defined cohorts of breast cancer patients and incorporate early changes in JAK/STAT pathway signatures during treatment as decision points to intensify or de-escalate therapy may be needed to realize the potential of these agents in clinical settings. In the long term, machine learning analyses applied to these multidimensional and multimodal datasets may generate clinically actionable “JAK/STAT-immunotherapy scores” that could drive the choice and timing of immune checkpoint blockade (PD-1/PD-L1 or CTLA-4 inhibitors, and potential others), selection of other combination partners, or identification of patients who should preferentially receive immunomodulatory strategies (e.g., IL-6/STAT3 blockade or metabolic reprogramming) before or alongside immune checkpoint inhibitors. Validating such JAK/STAT-based biomarker frameworks in different breast cancer subtypes, including HR^+^/HER2^-^ and HER2-positive disease, will be critical to harness the full potential of this pathway for personalizing immunotherapy in breast cancer. Longitudinal studies exploring the temporal dynamics of JAK/STAT signaling and PD-L1 expression during treatment will also be essential for refining therapeutic strategies.

Despite the promising landscape, several limitations remain. Many findings are based on preclinical models and require clinical validation. The potential toxicity associated with systemic JAK/STAT inhibition and the limited understanding of interactions between the pathway and other oncogenic drivers highlight the need for caution and further mechanistic studies. Nevertheless, with continued innovation and precision-guided intervention, targeting the JAK/STAT/PD-L1 axis stands as a transformative strategy to improve immunotherapeutic outcomes and usher in a new era of personalized breast cancer treatment.

## Availability of data and material

The original contributions presented in the study are included in the article.

## Acknowledgments

This work was supported by the 10.13039/100014718National Natural Science Foundation of China (No.82204504).

## Author contributions

H.S.: visualization, writing - original draft, writing - review & editing; X.Z.: project administration, supervision, writing - original draft, writing - review & editing.

## Declaration of interests

The authors have no conflicts of interest to declare.

## Declaration of generative AI and AI-assisted technologies in the writing process

While preparing this work, the authors used ChatGPT 4o by OpenAI to improve paper readability. After using this tool/service, the authors reviewed and edited the content as needed and took full responsibility for the publication’s content.
